# Hungry for Knowledge: Octopamine Signaling Regulates Hunger‐Enhanced Olfactory Learning

**DOI:** 10.1002/advs.202513842

**Published:** 2025-12-15

**Authors:** Huijuan Zhao, Guiyuan Shi, Ruixue Qin, Yinghao Sun, Wenbo Guo, Ruixia Shi, Minxian Peng, Jingxuan Yang, Jianjian Zhao, Qiuhan Liu, Jun Xiao, Ke Zhang, Qiang Liu, Wenxing Yang, He Liu

**Affiliations:** ^1^ Department of Systems Science Faculty of Arts and Sciences Beijing Normal University Zhuhai Guangdong 519000 China; ^2^ International Academic Center of Complex Systems Beijing Normal University Zhuhai Guangdong 519000 China; ^3^ School of Systems Science Beijing Normal University Beijing 100062 China; ^4^ Department of Physiology West China School of Basic Medical Sciences & Forensic Medicine Sichuan University Chengdu Sichuan 610041 China; ^5^ MOE Key Lab of Bioinformatics BNRIST Bioinformatics Division Department of Automation Tsinghua University Beijing 100084 China; ^6^ Department of Neuroscience City University of Hong Kong Tat Chee Avenue Kowloon Tong Hong Kong 610200 China; ^7^ Guangdong Institute of Intelligence Science and Technology Zhuhai Guangdong 510050 China; ^8^ Laboratory Safety and Equipment Management Office Beijing Normal University Zhuhai Guangdong 519000 China

**Keywords:** *C. elegans*, hunger, neural circuits, norepinephrine, octopamine, olfactory learning

## Abstract

Hunger, a state of nutrient deficiency, significantly enhances cognitive behaviors by heightening sensitivity to food‐related stimuli. However, the mechanisms by which hunger influences olfactory learning remain unclear. In this study, it is shown that aversive or appetitive memory is formed faster in hungry *C. elegans*. These findings reveal that hunger‐induced octopamine release from the interneuron RIC enhances both aversive and appetitive olfactory learning. By analyzing neural circuits downstream of RIC, two distinct pathways involved in memory formation are identified. For aversive learning, the sensory neuron ASH is activated via the *SER‐3* receptor, leading to glutamate release, which acts on the *GLR‐2* receptor in AIA interneuron during the starvation phase. During the training section, AIA is subsequently inhibited via the glutamate‐gated chloride channel *GLC‐3*. In contrast, octopamine targets AIY interneurons through the *SER‐6* receptor, promoting appetitive learning. Furthermore, it is indicated that norepinephrine, the mammalian homolog of octopamine, and alpha1‐adrenergic receptors may be involved in hunger‐enhanced olfactory learning in mice. These findings may offer insights into the neural mechanisms that underlie cognitive flexibility in response to physiological states.

## Graphical abstract

Researchers demonstrate that hunger state facilitates both aversive and appetitive olfactory learning. Two distinct octopamine signaling pathways are involved in aversive or appetitive memory formation in the hunger state. And, hunger state also facilitates the formation of both types of memories via an evolutionarily conserved norepinephrine (the mammalian homolog of octopamine) signaling pathway in mice.

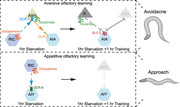

## 1 Introduction

Animals rely on learning to make informed decisions about their environment. For example, birds form appetitive learning to the visual cues around when hiding food, and retrieve food via memory‐guided navigation.^[^ ^1^, ^2^, ^3^ ^]^ Conversely, aversive memory, which involves negative, unpleasant, or harmful outcomes, helps animals enhance their chances of survival by steering clear of potentially dangerous substances.^[^ ^4^ ^]^ This type of memory is often strong and long‐lasting. For instance, *C. elegans* fed with the pathogenic bacteria *P. aeruginosa* generate an avoidance response to its odors specifically,^[^ ^5^, ^6^ ^]^ and this aversive memory may last several generations.^[^ ^7^, ^8^ ^]^ Hunger is a powerful motivator that influences various cognitive behaviors.^[^ ^9^ ^]^ Previous studies have demonstrated that hunger can enhance certain cognitive functions, such as attention and aggression, by relying on neuromodulators and peptides to alter neural responses.^[^ ^10^, ^11^ ^]^ Therefore, the activity of the underlying neural circuits should be changed to facilitate learning during the hunger state. Moreover, it is unclear whether the conserved neuromodulator induced by starvation is suitable for olfactory learning across species.

The mechanisms underlying hunger‐induced enhancements in cognitive performance are complex and multifaceted. Octopamine, a biogenic amine similar to norepinephrine in vertebrates, has been extensively studied in invertebrates. Octopamine is involved in a wide range of behaviors, including arousal, feeding, learning, and memory.^[^ ^12^, ^13^, ^14^, ^15^, ^16^ ^]^ In Drosophila, for example, octopamine has been shown to modulate both appetitive and aversive memories.^[^ ^17^, ^18^ ^]^ In *C. elegans*, starvation induces an increased octopamine biosynthesis to mediate various physiological and behavioral processes, including lipid metabolism, stress response, foraging and feeding behavior, etc.^[^ ^19^, ^20^, ^21^, ^22^ ^]^ In mammals, hunger regulates physiological events and behaviors through a variety of molecules, such as ghrelin, neuropeptide Y, agouti‐related peptide, etc.^[^ ^23^ ^]^ Among these molecules, norepinephrine plays a multifaceted role in regulating hunger and hunger‐related behaviors.^[^ ^24^, ^25^ ^]^ By integrating signals related to energy status, stress, and environmental cues, norepinephrine helps coordinate appropriate feeding behaviors to maintain energy homeostasis and respond to physiological needs.^[^ ^25^ ^]^ These studies indicate that octopamine and norepinephrine also integrate the internal states to regulate multiple behaviors.

In this study, we aim to elucidate the role of hunger in the formation of food‐deprivation‐associated aversive and food‐presentation‐associated appetitive memories in two model organisms, *C. elegans* and mice. By leveraging the simplicity of the *C. elegans* nervous system and the complexity of mammalian behaviors, we seek to uncover conserved mechanisms that underlie hunger‐enhanced olfactory learning across species. We demonstrated the essential role of octopamine signaling in facilitating both aversive olfactory learning and appetitive olfactory learning in *C. elegans*. Through the dissection of neural circuits downstream of octopaminergic neurons, we have pinpointed two discrete neural pathways that mediate hunger‐induced improvements in aversive olfactory learning and appetitive olfactory learning, respectively. Additionally, we explored the parallels in mice and found that norepinephrine levels increased after two days of starvation. Artificially elevating norepinephrine mimicked hunger‐facilitated aversive and appetitive learning, while blocking alpha‐adrenergic receptors attenuated this effect. These results suggest a potentially evolutionarily conserved mechanism underlying hunger‐induced behavioral modulation. Our findings contribute to a deeper understanding of how hunger modulates memory formation and the neural substrates involved. This knowledge not only advances the field of neuroscience but also has potential implications for developing strategies to enhance memory and learning in various contexts, including educational and clinical settings.

## 2 Results

### 2.1 Starvation Induces a Distinct Physiological State in *C. Elegans*

To confirm that worms enter a distinct physiological state after starvation, we quantified the levels of 537 metabolites (Table S1, Supporting Information), including those involved in energy metabolism, fatty acids, amino acids, neurotransmitters, etc., under satiated, 1 h starvation, and 3 h starvation conditions. Indeed, principal component analysis results revealed that the 1 and 3 h starvation groups were largely intermingled and distinctly separated from the satiated worms (Figure S1A, Supporting Information). Pearson correlation analysis and hierarchical clustering results showed an overall trend in which most within‐group pairs exhibited positive correlations, while the majority of comparisons between satiated and starved worms showed an anti‐correlation (Figure S1B, Supporting Information).

Taking satiety, 1 h starvation, and 3 h starvation as individual points along a continuous starvation process, we systematically analyzed the changes in different metabolites and utilized ANOVA (Analysis of Variance) with time points as the independent variable for each metabolite, followed by the Benjamini‐Hochberg procedure to control the false discovery rate (FDR) of multiple hypothesis tests across metabolites. We observed that the metabolites with statistically significant changes (adjusted *p*‐value < 0.05) exhibited trends of continuous increase, continuous decrease, a pattern of decrease followed by increase, and a pattern of increase followed by decrease (Figure S1C–F, Supporting Information).

To further understand the biological significance of the differentially regulated metabolites during starvation, we performed KEGG pathway enrichment analysis, with a particular focus on the “Metabolism” category, as most of the altered metabolites were enriched in this pathway class (Figure S1G–J, Supporting Information). In addition, tryptophan‐derived metabolites play a critical role in the metabolic response to starvation. Tryptophan is metabolized mainly through three pathways: the kynurenine (Kyn) pathway, the serotonin (5‐HT) pathway, and the indole pathway.^[^ ^26^ ^]^ During starvation, the levels of kynurenic acid and indole‐3‐carboxaldehyde, two key metabolites derived from tryptophan, progressively declined, suggesting that tryptophan metabolism is highly sensitive to nutritional deficiency and rapidly adjusted to energy stress. This observation is consistent with previous reports showing that starvation accelerates kynurenic acid degradation in *C. elegans*.^[^ ^27^ ^]^ Beyond tryptophan metabolism, branched‐chain amino acids (BCAAs) also showed dynamic changes in response to nutrient deprivation. BCAAs are essential amino acids that function not only as building blocks of proteins but also as critical regulators of energy homeostasis. Among them, L‐leucine displayed a biphasic pattern: its levels decreased after 1 h of starvation but rebounded after 3 h. This trend may reflect a two‐step metabolic strategy, in which leucine is first catabolized to meet immediate energy demand, followed by a metabolic shift that enhances BCAA utilization as a primary energy source during prolonged starvation.^[^ ^28^ ^]^ Notably, glutathione plays a crucial role in regulating the glutamatergic neurotransmission^[^ ^29^ ^]^ and is involved in the modulation of aversive learning processes.^[^ ^6^ ^]^ Taken together, these results suggest that starvation‐induced alterations in amino acid and glutamate‐related metabolism may not only reshape energy homeostasis but also influence subsequent learning processes.

### 2.2 Hunger State Facilitates Subsequential Aversive Olfactory Learning

**Figure 1:**
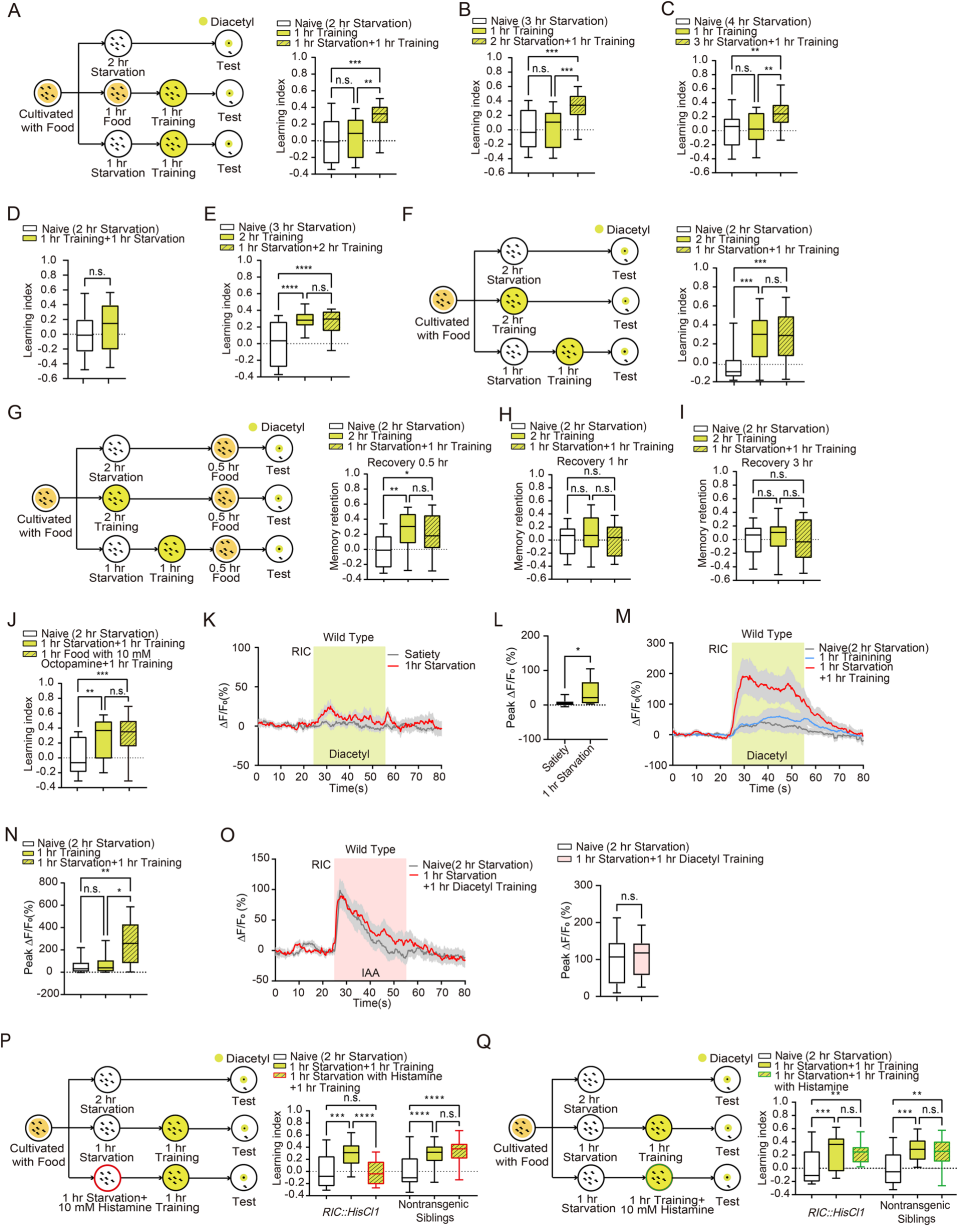
Octopamine signaling from the RIC neuron enhances the formation of food‐deprivation‐associated aversive olfactory memory. A–C) Schematic representation of the behavioral paradigm for aversive olfactory learning. The aversive learning index was assessed in naive animals, those immediately after 1 h training, and those after 1 h training followed by 1 h A) (*n* = 22 per group), 2 h B) (*n* = 22 per group) or 3 h C) (*n* = 21 per group) starvation. D) Statistical comparison of the aversive learning index between worms subjected to 1 h starvation followed by 1 h training and control animals subjected to 2 h starvation (*n* = 24 per group). E) Statistical comparison of the aversive learning index among worms subjected to 2 h training followed by 1 h starvation (*n* = 21), naive animals (*n* = 22), and worms subjected to 2 h training alone (*n* = 22). F) Schematic representation of the behavioral paradigm and statistical comparison of the aversive learning index among worms subjected to 1 h training followed by 1 h starvation (*n* = 25), naive animals (*n* = 27), and those subjected to 2 h training alone (*n* = 27). G–I) Schematic representation of the behavioral paradigm for memory retention after 0.5 h recovery with food. Statistical comparison of memory retention at 0.5 h G) (Naive, *n* = 23; 2h Training, *n* = 21; 1 h Starvation + 1 h Training, *n* = 22), 1 h H) (Naive, *n* = 24; 2 h Training, *n* = 23; 1 h Starvation + 1 h Training, *n* = 22), and 3 h I) (Naive, *n* = 25; 2 h Training, *n* = 24; 1 h Starvation + 1 h Training, *n* = 24) of recovery following 1 h training after 1 h starvation and 2 h training. (J) Aversive learning index comparison between worms subjected to 1 h training followed by 1 h starvation and those subjected to 1 h training followed by exogenous octopamine treatment with food for 1 h (*n* = 24 per group). K) Traces of *GCaMP6* signals in the RIC neuron in response to diacetyl under satiated and 1 h starvation conditions. Colored lines and light gray areas represent mean values and SEM, respectively. L) Statistical comparison of peak *GCaMP6* signals in RIC during diacetyl exposure under satiated and 1 h starvation conditions (*n* = 13 per group). M) Traces of *GCaMP6* signals in the RIC neuron in response to diacetyl under naive, 1 h training, and 1 h training followed by 1 h starvation conditions. Colored lines and light gray areas represent mean values and SEM, respectively. N) Statistical comparison of peak *GCaMP6* signals in RIC during diacetyl exposure under naive, 1 h training, and 1 h training followed by 1 h starvation conditions (*n* = 16 per group). O) Traces (Left) and statistical comparison (Right) of *GCaMP6* signals in the RIC neuron in response to butanone under naive (*n* = 11) and 1 h training followed by 1 h starvation conditions (*n* = 10). Colored lines and light gray areas represent mean values and SEM, respectively. P) Schematic representation (Left) and statistical comparison (Right) of the behavioral paradigm for manipulating the RIC neuron with histamine treatment during the starvation session (*n* = 23 per group). Q) Schematic representation (Left) and statistical comparison (Right) of the behavioral paradigm for manipulating the RIC neuron with histamine treatment during the training session. Data are presented as boxplots showing the maximum value, 75th percentile, median, 25th percentile, and minimum value (*n* = 22 per group). Statistical significance was determined using Student's t‐test or two‐way ANOVA with Tukey's post hoc test. ^*^ *p* < 0.05; ^**^ *p* < 0.01; ^***^ *p* < 0.001; ^****^ *p* < 0.0001; n.s., not significant.

Previous studies have established an aversive olfactory learning paradigm in *C. elegans*.^[^ ^30^ ^]^ Building on this protocol, we exposed 1‐day‐old adult worms to an empty Nematode Growth Medium (NGM) plate coated with 100 µL of 1:10000 diluted diacetyl for various durations to establish an association between diacetyl and the absence of food. We then assessed the chemotaxis towards diacetyl using the navigation index.^[^ ^5^, ^6^ ^]^ Briefly, the navigation index was calculated as the cosine of the angle between the worm's movement direction and its target direction, with higher values indicating greater target attractiveness.^[^ ^5^ ^]^ We then calculated the learning index by normalizing the navigation index to that of the naive group, with higher values indicating a stronger learning effect (Experimental Section, Figure S2A,C,D,F,G, Supporting Information). These two parameters describe general features of the chemotaxis trajectory and are therefore not influenced by the worm's locomotion speed. And we measured the speed of the worms on an empty NGM plate in the absence of OP50 (a strain of *Escherichia coli*). Indeed, we found that diacetyl association with starvation did not affect locomotion speed, (Figure S2B,D,F,H, Supporting Information); however, it did alter the attractiveness of diacetyl (Figure S2C,E,G, Supporting Information). We found that a 2 h association was the minimum time required for worms to develop aversive memory towards diacetyl (Figure S2C,E,G, Supporting Information), whereas a 1 h training session induced a learning index comparable to that of the naive group (**Figure** **1**A; Figure S2A, Supporting Information). To examine the concentration dependency during training in aversive diacetyl learning, we varied the diacetyl concentration during the training session while keeping it consistent during the testing session. As shown in Figure S2I, Supporting Information worms starved for 1 h in the presence of 100 µL of 1:1000 diluted diacetyl exhibited a significantly higher learning index compared to the naive group. The result indicates that the aversive memory is formed after 1 h of training with 1:1000 diluted diacetyl.

To test whether the hunger state modulates subsequent aversive olfactory learning, we tested how additional starvation before the training session affects the learning index. We starved worms for 1 h before 1 h training, while worms starved for 2 h or trained for 1 h alone served as control groups for naive and standard training, respectively. Notably, the learning index of worms subjected to 1 h starvation followed by 1 h training was significantly higher than that of the naive group and worms with only 1 h training (Figure 1A; Figure S3A, Supporting Information). Similar enhancements in learning index were observed in worms subjected to 2 or 3 h of starvation before the 1 h training session (Figure 1B,C; Figure S3B,C, Supporting Information), indicating a non‐linear enhancement of olfactory learning by hunger state. To investigate whether the sequence of starvation and training affects learning, we performed experiments in which worms were starved after training. Interestingly, 1 h of starvation following a 1 h training session did not alter the learning index (Figure 1D; Figure S3D, Supporting Information). These data suggest that a state of hunger facilitates subsequent food‐deprivation‐associated aversive olfactory learning.

Having established that the hunger state promotes aversive olfactory learning, we next queried whether hunger influences the level of learning. The worms underwent a 2 h training session, during which they formed an aversive memory toward diacetyl (Figure S2C, Supporting Information). The comparable learning indexes of worms with 1 h starvation followed by 2 h training and those of 2 h training alone indicate that additional starvation does not enhance the learning level (Figure 1E; Figure S3E, Supporting Information).

Both 2 h training and 1 h starvation followed by 1 h training induced aversive memory (Figure 1A; Figure S2C, Supporting Information), we then compared the learning indexes between these two protocols. The learning indexes for worms subjected to 2 h training and those with 1 h starvation followed by 1 h training were found to be similar (Figure 1F; Figure S3F, Supporting Information). We further examined memory retention after worms were returned to plates with food and found that the forgetting rates for memories induced by these two protocols were comparable (Figure 1G–I; Figure S3G–I, Supporting Information). These results indicate that 1 h starvation followed by 1 h training yields learning effects analogous to those of 2 h training.

### 2.3 An Octopamine Neural Circuit Mediates Hunger‐State‐Enhanced Aversive Olfactory Learning

Octopamine is released in response to stimuli associated with food scarcity, modulating behaviors to enhance survival during nutrient deprivation.^[^ ^19^, ^31^ ^]^ To characterize the role of octopamine in regulating hunger‐state‐enhanced aversive olfactory learning, we administered a mixture of 100 µL of 10 mm octopamine with food to the worms 1 h before the 1 h training session. The comparable learning indexes were observed in worms that received octopamine with food and those subjected to 1 h starvation followed by 1 h training (Figure 1J; Figure S3J, Supporting Information). To further confirm the role of octopamine, we examined the learning effect in *tbh‐1* mutants, in which octopamine synthesis was impaired, consistently, worms subjected to 1 h starvation followed by 1 h training showed similar learning index and speed with those subjected to 1 h training (Figure S4A,B, Supporting Information). Interestingly, octopamine does not appear to be involved in the learning process, as evidenced by the significantly increased learning index in *tbh‐1* mutants after 2 h of training (Figure S4C, Supporting Information). Interestingly, 2 h training reduced locomotion speed in *tbh‐1* mutants (Figure S4D, Supporting Information). Above all, this result suggests that octopamine is involved in hunger‐facilitated aversive olfactory learning.

Given that the interneuron RIC is the sole source of octopamine in *C. elegans*,^[^ ^32^ ^]^ we investigated RIC's response to diacetyl to clarify the involvement of octopamine signaling. Transgenic animals expressing *GCaMP6* specifically in RIC were placed in a microfluidic system,^[^ ^33^ ^]^ and *GCaMP6* intensity dynamics were imaged during a 30 s diacetyl presentation. Interestingly, RIC showed no response to diacetyl under satiated conditions, whereas after 1 h of starvation, RIC displayed significant activation upon diacetyl presentation (Figure 1K,L). RIC exhibited a significantly increased response to diacetyl under 1 h starvation followed by a 1 h training condition, in comparison with those under naive and 1 h training alone conditions (Figure 1M,N). Next, we measured the calcium response to isoamyl alcohol (IAA) to determine whether the training effects on RIC activity are odor‐specific or represent a general effect across different odors. We found that 1 h of starvation following 1 h of diacetyl training did not alter the RIC response to IAA, suggesting that the training effects on RIC activity are odor‐specific (Figure 1O). Since fluid switching in the microfluidic system may generate shear forces,^[^ ^34^ ^]^ we performed a buffer‐to‐buffer switch while recording RIC activity. We found that RIC did not respond to the buffer‐to‐buffer switch in our microfluidic system (Figure S5A, Supporting Information).

To explore the underlying mechanism by which the RIC neuron is sensitive to the olfactory stimulus after starvation, we performed electrophysiological recordings on RIC neurons from satiated and starved animals. Surprisingly, the resting membrane potential of RIC neurons was not altered by starvation (Figure S6A, Supporting Information). Under the current‐clamp configuration, current injection steps elicited similar membrane potential changes in RIC from both groups (Figure S6B, Supporting Information). These results suggest that the activation of RIC by diacetyl in starved animals, as observed in calcium imaging, is unlikely to be attributed to membrane potential changes associated with voltage‐gated calcium channels. Notably, under voltage‐clamp conditions, whole‐cell currents were slightly increased in starved animals for both peak and steady‐state currents (Figure S6C,D, Supporting Information). It is plausible that the hunger state induces the increased expression of voltage‐gated potassium channels or their increased trafficking to the membrane surface in RIC. However, the identity of these channels and their connection to the heightened excitability of RIC remain unclear.

To establish a causal link between RIC activity and hunger‐state‐enhanced aversive olfactory learning, we introduced *HisCl1* (*histamine‐gated chloride channel 1*) specifically into RIC and treated animals with histamine during 1 h starvation to inhibit RIC. Taking advantage of its dynamics on the scale of seconds, *HisCl1* has been a widely used tool in the *C. elegans* field to study neuronal functions related to behavior by temporarily inhibiting specific neuronal activity.^[^ ^6^, ^35^, ^36^, ^37^, ^38^ ^]^ The learning index of transgenic animals treated with histamine during the starvation period was significantly reduced compared to untreated controls, while nontransgenic siblings showed no difference in learning index with or without histamine treatment (Figure 1P; Figure S3K, Supporting Information). In contrast, inhibiting RIC during the training period did not affect hunger state‐related aversive olfactory learning enhancement (Figure 1Q; Figure S3K, Supporting Information). These findings suggest that octopamine release from the activated RIC neuron during starvation establishes a state that enables rapid aversive learning to adapt to olfactory cues, indicating a distinct neural circuit for hunger‐facilitated learning compared to regular learning processes.

**Figure 2:**
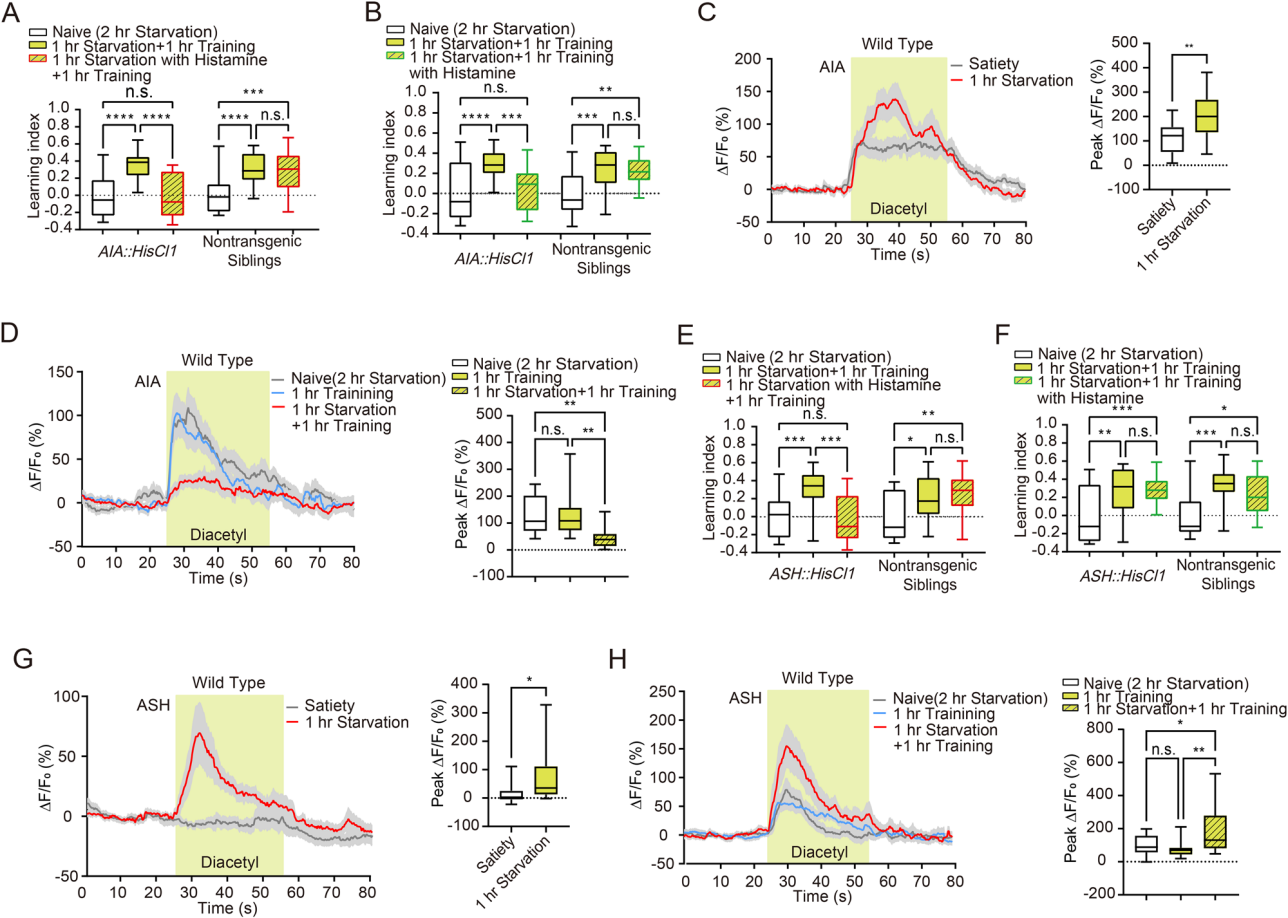
Neurons AIA and ASH regulate hunger‐enhanced food‐deprivation‐associated aversive olfactory learning. A,B) Inhibition of neuron AIA during either the 1 h starvation session A) or the 1 h training session (*n* = 23 per group) B) abolishes hunger‐enhanced aversive olfactory learning (*n* = 23 per group). C) Left: Traces of *GCaMP6* signals in the AIA neuron in response to diacetyl under satiated (*n* = 13) and 1 h starvation conditions (*n* = 14). Colored lines and light gray areas represent mean values and SEM, respectively. Right: Statistical comparison of peak *GCaMP6* signals in AIA during diacetyl exposure under satiated and 1 h starvation conditions. D) Left: Traces of *GCaMP6* signals in the AIA neuron in response to diacetyl under naive, 1 h training, and 1 h training followed by 1 h starvation conditions (*n* = 15 per group). Colored lines and light gray areas represent mean values and SEM, respectively. Right: Statistical comparison of peak *GCaMP6* signals in AIA during diacetyl exposure under naive, 1 h training, and 1 h training followed by 1 h starvation conditions. E) Inhibition of neuron ASH during the 1 h starvation session abolishes hunger‐enhanced aversive olfactory learning (*n* = 23 per group). F) Inhibition of neuron ASH during the 1 h training session does not affect hunger‐enhanced aversive olfactory learning (*n* = 21 per group). G) Left: Traces of *GCaMP6* signals in the ASH neuron in response to diacetyl under satiated (*n* = 15) and 1 h starvation conditions (*n* = 14). Colored lines and light gray areas represent mean values and SEM, respectively. Right: Statistical comparison of peak *GCaMP6* signals in ASH during diacetyl exposure under satiated and 1 h starvation conditions. (H) Left: Traces of *GCaMP6* signals in the ASH neuron in response to diacetyl under naive (*n* = 16), 1 h training (*n* = 15), and 1 h training followed by 1 h starvation conditions (*n* = 16). Colored lines and light gray areas represent mean values and SEM, respectively. Right: Statistical comparison of peak GCaMP6 signals in ASH during diacetyl exposure under naive, 1 h training, and 1 h training followed by 1 h starvation conditions. Data are presented as boxplots showing the maximum value, 75th percentile, median, 25th percentile, and minimum value. Statistical significance was assessed using Student's *t*‐test or two‐way ANOVA with Tukey's post hoc test. ^*^ *p* < 0.05; ^**^ *p* < 0.01; ^***^ *p* < 0.001; ^****^ *p* < 0.0001; n.s., not significant.

Interneuron AIA has been identified as a key player in starvation‐related olfactory learning.^[^ ^30^, ^39^ ^]^ To investigate how enhanced octopamine signals from RIC participate in the subsequent learning and to dissect the underlying neural circuit for hunger state‐facilitated aversive olfactory learning, we generated transgenic animals with *HisCl1* specifically expressed in AIA. Inhibiting AIA activity during a 1 h starvation period using histamine treatment in *AIA::HisCl1* animals, followed by a 1 h training session, resulted in a significantly decreased learning index compared to untreated transgenic controls, indicating that AIA inhibition during prior starvation abolishes the hunger‐facilitated aversive learning effect (Figure 2A; Figure S3L, Supporting Information). We found that inhibiting AIA activity during the training session also attenuated the enhanced learning, evidencing by the decreased learning index of transgenic worms with histamine treatment during the 1 h training (**Figure** **2**B; Figure S3O, Supporting Information). These results indicate that AIA participates in both the prior starvation and subsequent training sessions, facilitating hunger‐induced aversive olfactory learning. Calcium imaging of AIA's response to diacetyl revealed increased calcium responses after 1 h of starvation, compared to satiated conditions (Figure 2C). Interestingly, AIA's response to diacetyl decreased after 1 h of starvation followed by 1 h of training (Figure 2D). Previous studies have shown that decreased AIA activity regulates the aversive memory of diacetyl.^[^ ^40^, ^41^ ^]^ Consistently, our study found that 1 h of starvation followed by 1 h of training also reduces AIA activity, suggesting that this reduction may underlie aversive memory formation in our training protocol. As a control experiment, we examined AIA response to a buffer‐to‐buffer switch and found that AIA did not respond to shear stress in our microfluidic system (Figure S5C, Supporting Information).

Our results suggest that the AIA neuron acts as a downstream neuron of RIC for hunger‐facilitated aversive olfactory learning. The relationship between RIC and AIA at the circuit level could be direct or mediated by an intermediate neuron. Connectome reconstruction studies showed no direct synapses between RIC and AIA^[^ ^42^, ^43^ ^]^ and Single cell RNAseq results showed that AIA expresses low levels of octopamine receptors.^[^ ^44^ ^]^ These pieces of evidence support an intermediate neuron between RIC and AIA neurons. We identified neuron ASH as a top candidate as the intermediate neuron due to its high expression of the octopamine receptor *SER‐3* and strong synaptic connection with AIA.^[^ ^42^, ^43^, ^44^ ^]^ We generated transgenic animals expressing *HisCl1* in ASH neurons and examined ASH's role in hunger‐facilitated aversive olfactory learning. Inhibiting ASH during the 1 h starvation period indeed attenuated hunger‐facilitated aversive olfactory learning (Figure 2E; Figure S3 M, Supporting Information). While inhibiting ASH during the training session resulted in a similar learning index to animals without ASH activity manipulation (Figure 2F; Figure S3P, Supporting Information), suggesting that ASH regulates hunger‐facilitated aversive olfactory learning during the starvation period but not the training session, similar to RIC function (Figure 1P,Q). We imaged ASH's calcium response to diacetyl under satiety or 1 h starvation conditions to dissect ASH's function in hunger‐induced aversive olfactory learning facilitation. Interestingly, similar to the neuron RIC, ASH did not respond to diacetyl under satiety conditions but generated robust calcium activity during diacetyl presentation under starvation conditions (Figure 2G). The response to diacetyl in ASH significantly increased after 1 h of starvation followed by 1 h of training, compared to those under 1 h of training alone or naive conditions (Figure 2H). Our results showed that 1 h of starvation may cause a nonsignificant increase in ASH calcium activity in response to the buffer‐to‐buffer switch in our microfluidic system (Figure S5B, Supporting Information). It is well established that the ASH neuron, as a major nociceptive neuron, plays a key role in generating avoidance behaviors.^[^ ^45^, ^46^, ^47^ ^]^ In our study, we found that ASH responds to diacetyl after 1 h of starvation, and its response is enhanced after 1 h of starvation followed by 1 h of training. This increased ASH activity may contribute to the formation of aversive memory in our training protocol.

Based on the above results, we identified a neural circuit, RIC → ASH → AIA, that mediates hunger‐facilitated sequential aversive olfactory learning. In this circuit, RIC and ASH function primarily during the starvation phase, while AIA is involved in both the starvation and training phases.

### 2.4 Octopamine Receptor *SER‐3*, and AMPA Receptors *GLR‐2, GLC‐3* Regulate the Aversive Olfactory Learning

**Figure 3:**
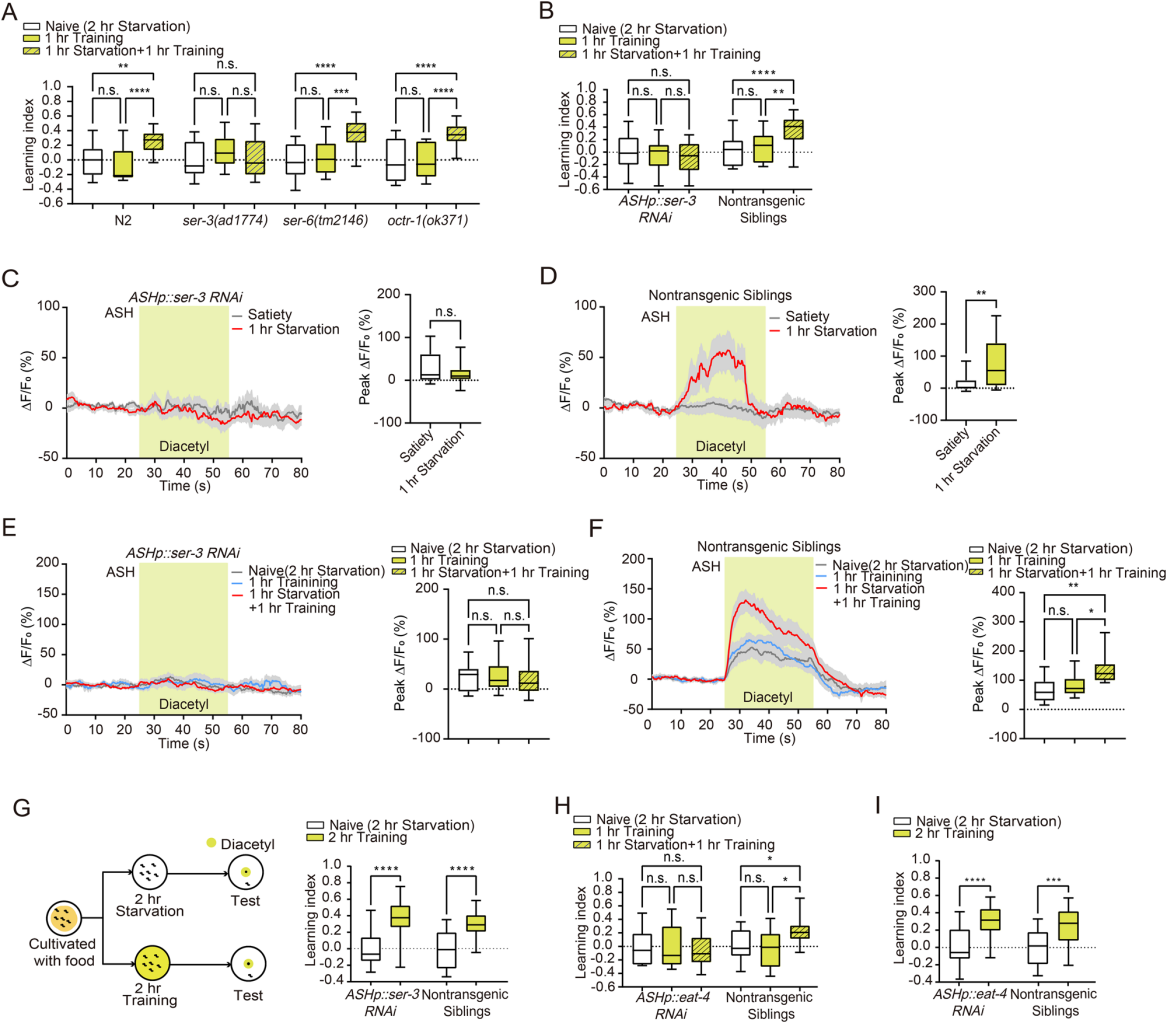
Octopamine receptor SER‐3 in ASH regulates hunger‐enhanced food‐deprivation‐associated aversive olfactory learning. A) Statistical comparison of the aversive learning index among wild‐type (*n* = 20 per group), ser‐3 (*n* = 20 per group), ser‐6 (*n* = 18 per group), and octr‐1 (Naive, *n* = 19; 1 h Training, *n* = 19; 1 h Starvation + 1 h Training, *n* = 17) loss‐of‐function worms subjected to 1 h training followed by 1 h starvation, naive animals, and those subjected to 1 h training alone. B) Silencing ser‐3 expression in ASH abolishes hunger‐enhanced aversive olfactory learning (*n* = 23 per group). C,D) Silencing ser‐3 expression in ASH abolishes the ASH neuron's response to diacetyl odor (Satiety, *n* = 15; 1 h Starvation, *n* = 14). E,F) Silencing ser‐3 expression in ASH affects ASH's response to diacetyl odor following aversive learning (E: Naive, *n* = 15; 1 h Training, *n* = 14; 1 h Starvation + 1 h Training, *n* = 15; F: *n* = 10 per group). G) Left: Schematic representation of the 2 h training paradigm. Right: Statistical comparison of the aversive learning index between naive animals and the 2 h training group (*n* = 21 per group). H) Silencing eat‐4 expression in ASH abolishes hunger‐enhanced aversive olfactory learning (*ASHp::eat‐4 RNAi*: *n* = 23 per group; Nontransgenic Siblings: Naive, *n* = 20; 1 h Training, *n* = 20; 1 h Starvation + 1 h Training, *n* = 21). I) Silencing eat‐4 expression in ASH does not affect aversive memory formation after 2 h training (*ASHp::eat‐4 RNAi*: *n* = 23 per group; Nontransgenic Siblings: *n* = 22 per group). Data are presented as boxplots showing the maximum value, 75th percentile, median, 25th percentile, and minimum value. Statistical significance was determined using Student's *t*‐test or two‐way ANOVA with Tukey's post hoc test. ^*^ *p* < 0.05; ^**^ *p* < 0.01; ^***^ *p* < 0.001; ^****^ *p* < 0.0001; n.s., not significant.

Next, we aimed to identify the neurotransmitters and receptors involved in this circuit. In *C. elegans*, three octopamine receptors have been identified: *SER‐3*, *SER‐6*, and *OCTR‐1*.^[^ ^31^, ^48^, ^49^, ^50^, ^51^, ^52^ ^]^ We examined the role of each receptor by performing behavioral assays using loss‐of‐function mutants. Results showed that hunger‐facilitated aversive olfactory learning was attenuated in *ser‐3* mutants, while *ser‐6* and *octr‐1* mutants exhibited similar behavioral outputs to the N2 wild‐type (**Figure** **3**A; Figure S7A, Supporting Information), suggesting that *SER‐3* is involved in hunger‐facilitated aversive olfactory learning. These results, along with the high expression level of *ser‐3* ^[^ ^44^ ^]^ and its functional role in ASH,^[^ ^48^ ^]^ suggest that *SER‐3* in ASH neurons may mediate hunger‐facilitated aversive olfactory learning. To examine this hypothesis, we generated transgenic worms with reduced *ser‐3* expression specifically in ASH using RNAi, and tested their learning ability. We found that *ASH::ser‐3 RNAi* worms displayed similar learning indexes under naive, 1 h training alone, and 1 h starvation followed by 1 h training conditions. In contrast, non‐transgenic siblings exhibited an enhanced learning index when starved for 1 h and then trained for 1 h (Figure 3B; Figure S7B, Supporting Information). To further confirm the necessity of *ser‐3* in ASH, we examined the learning effect in *ASH::ser‐3 RNAi* worms after 1 h octopamine administration followed by 1 h of training. We found that *ASH::ser‐3 RNAi* worms failed to generate a learning effect (Figure S8A, Supporting Information), whereas wild‐type worms showed a significant learning index (Figure 1J). ASH neuron in *ASH::ser‐3 RNAi* worms displayed no response to diacetyl after 1 h starvation (Figure 3C), while non‐transgenic siblings exhibited a strong response (Figure 3D). ASH neurons in *ASH::ser‐3 RNAi* worms exhibited no response to diacetyl under naive, 1 h training alone, and 1 h starvation followed by 1 h training conditions (Figure 3E). The response to diacetyl in non‐transgenic siblings was significantly enhanced after 1 h of starvation followed by 1 h of training, compared with worms subjected to 1 h of training alone or naïve conditions (Figure 3F). These results indicate the crucial role of *SER‐3* in ASH in hunger‐induced aversive olfactory learning facilitation. Given that ASH neurons function during the starvation period for hunger‐facilitated aversive olfactory learning (Figure 2E–H), we examined the role of *SER‐3* in ASH for regular aversive olfactory learning. We trained *ASH::ser‐3 RNAi* worms for 2 h and tested their chemotaxis to diacetyl immediately after training. The significantly increased learning index indicates that *SER‐3* in ASH is not involved in regular aversive olfactory learning (Figure 3G; Figure S7C, Supporting Information).

We have found that ASH responds to octopamine signals from RIC through the octopamine receptor *SER‐3* for hunger‐facilitated aversive olfactory learning. Next, we investigated the signals from ASH to AIA. Previous studies have shown that ASH releases glutamate.^[^ ^45^, ^47^ ^]^ Therefore, we interrupted glutamatergic neurotransmission to examine the role of glutamate signaling in ASH for hunger‐facilitated aversive olfactory learning. EAT‐4 encodes a vesicular glutamate transporter that loads glutamate into synaptic vesicles, thus playing a key role in glutamatergic transmission.^[^ ^53^ ^]^ We generated *ASH::eat‐4 RNAi* worms and examined their learning behaviors. We found that *ASH::eat‐4 RNAi* animals revealed similar learning index under naive, 1 h training, and 1 h starvation followed by 1 h training conditions (Figure 3H; Figure S7D, Supporting Information). However, *ASH::eat‐4 RNAi* animals displayed normal learning ability under the 2 h training condition (Figure 3I; Figure S7E, Supporting Information). These results indicate that glutamate output from ASH regulates hunger‐induced learning facilitation, but not regular aversive olfactory learning.

**Figure 4:**
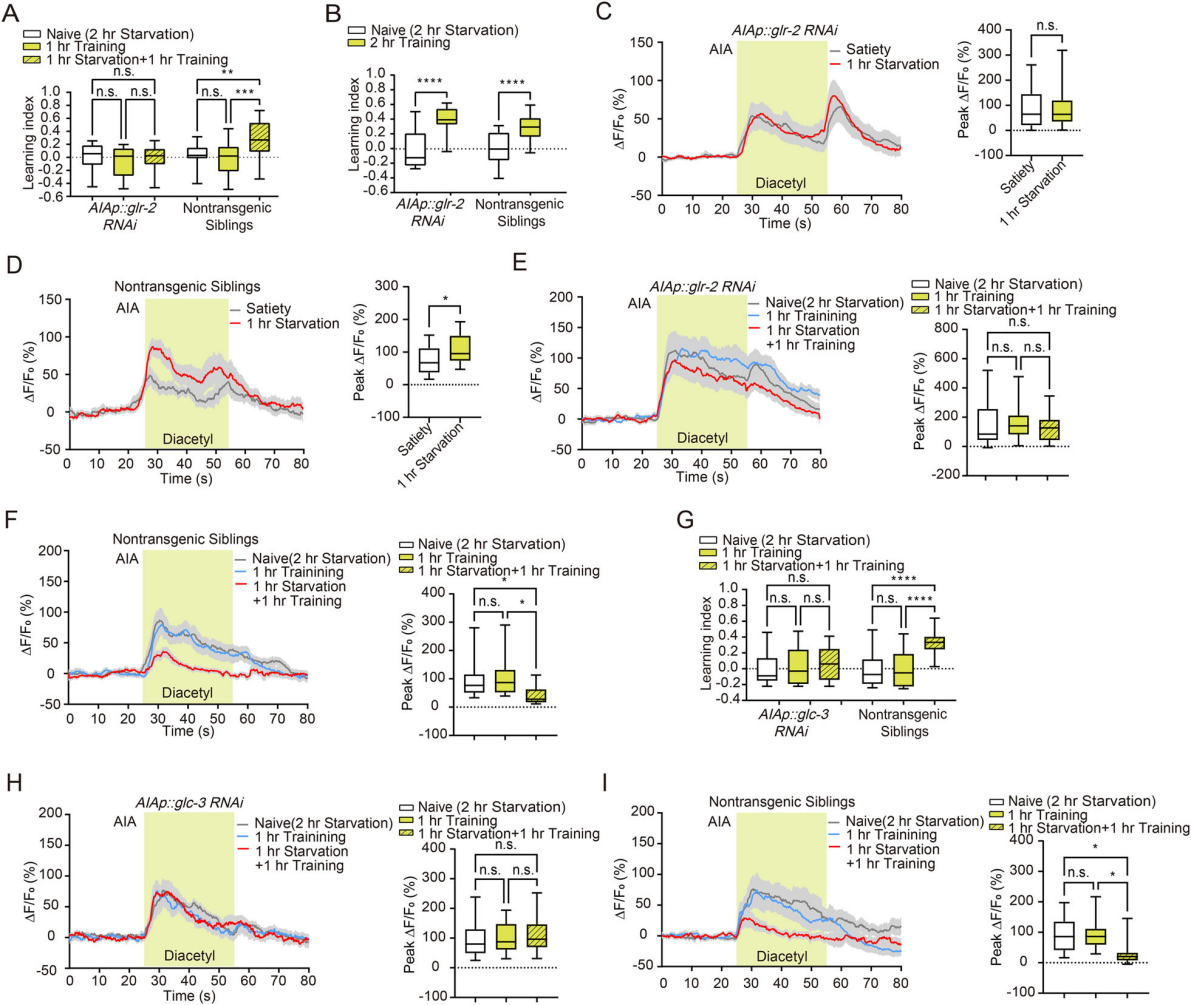
Glutamate receptors GLR‐2 and GLC‐3 in AIA regulate hunger‐enhanced food‐deprivation‐associated aversive olfactory learning. A) Silencing glr‐2 expression in AIA abolishes hunger‐enhanced aversive olfactory learning (*AIAp::glr‐2 RNAi*: Naive, *n* = 22; 1 h Training, *n* = 23; 1 h Starvation + 1 h Training, *n* = 23; Nontransgenic Siblings: *n* = 23 per group). B) Silencing glr‐2 expression in AIA does not affect aversive memory formation after 2 h training (*n* = 23 per group). C,D) Silencing glr‐2 expression in AIA abolishes starvation‐induced changes in AIA's response to diacetyl (*AIAp::glr‐2 RNAi*: Satiety, *n* = 25; 1 h Starvation, *n* = 24; Nontransgenic Siblings: Satiety, *n* = 14; 1 h Starvation, *n* = 24). E,F) Silencing glr‐2 expression in AIA abolishes changes in AIA's response to diacetyl after 1 h training followed by 1 h starvation (E: Naive, *n* = 20; 1 h Training, *n* = 19; 1 hr Starvation + 1 h Training, *n* = 21; F: Naive, *n* = 15; 1 h Training, *n* = 15; 1 h Starvation + 1 h Training, *n* = 17). G) Silencing glc‐3 expression in AIA abolishes hunger‐enhanced aversive olfactory learning (*AIAp::glc‐3 RNAi*: *n* = 23 per group; Nontransgenic Siblings: *n* = 22 per group). H,I) Silencing glc‐3 expression in AIA abolishes changes in AIA's response to diacetyl after 1 h training followed by 1 h starvation (H: Naive, *n* = 12; 1 h Training, *n* = 12; 1 h Starvation + 1 h Training, *n* = 14; I: Naive, *n* = 10; 1 h Training, *n* = 7; 1 h Starvation + 1 h Training, *n* = 11). Data are presented as boxplots showing the maximum value, 75th percentile, median, 25th percentile, and minimum value. Statistical significance was determined using Student's *t*‐test or two‐way ANOVA with Tukey's post hoc test. ^*^ *p* < 0.05; ^**^ *p* < 0.01; ^***^ *p* < 0.001; ^****^ *p* < 0.0001; n.s., not significant.

Next, we sought to identify the glutamate receptors in neuron AIA. Taylor et al. showed that *glr‐2* is highly expressed in AIA^[^ ^44^ ^]^ and mediates several behaviors, such as arousal.^[^ ^54^ ^]^ We knocked down *glr‐2* expression in AIA using RNAi and examined its role in hunger‐facilitated aversive olfactory learning. We found that 1 h starvation followed by 1 h training generated a similar learning index to the naive and 1 h training conditions in *AIA::glr‐2 RNAi* animals (**Figure** **4**A; Figure S7F, Supporting Information). However, *GLR‐2* in AIA did not participate in 2 h training (Figure 4B; Figure S7G, Supporting Information). These results indicate that glutamate signaling between ASH and AIA specifically regulates hunger‐induced aversive olfactory learning facilitation, but not regular aversive learning. We imaged AIA's calcium events in *glr‐2* knocked‐down background, and indeed, *GLR‐2* in AIA regulates AIA's response to diacetyl after 1 h starvation and 1 h starvation followed by 1 h aversive learning (Figure 4C–F).

We found that AIA's calcium responses were significantly reduced after 1 h of starvation followed by 1 h of aversive learning (Figure 2D). This phenomenon cannot be explained by the excitatory glutamate receptor *GLR‐2* on AIA, although *GLR‐2* may mediate signal transmission between ASH and AIA during the starvation period. Therefore, we hypothesize that an inhibitory glutamate receptor is involved during the learning phase. A previous study showed that AIA is activated by the sensory neuron AWA through gap junctions, while being inhibited by other sensory neurons via glutamatergic signaling.^[^ ^40^ ^]^ Notably, the inhibitory glutamate receptor gene *glc‐3* is highly expressed in AIA.^[^ ^44^ ^]^ Therefore, we investigated the role of *glc‐3* in AIA during hunger‐facilitated learning by assessing the learning index and calcium response of *AIA::glc‐3 RNAi* worms. The results showed that *AIA::glc‐3 RNAi* worms exhibited similar learning indexes (Figure 4G) and calcium activity levels (Figure 4H,I) across the 1 h starvation followed by 1 h training group, the 1 h learning‐only group, and the naive group. These findings suggest that *glc‐3* in AIA is involved in the hunger‐facilitated learning process.

Based on all the results, we have delineated a circuit, RIC → ASH → AIA, that regulates hunger‐induced aversive olfactory learning facilitation, where the octopamine signal transduction between RIC and ASH neuron functions during the starvation period, and the AIA neuron is involved in both the starvation and training phases (Figure 6H). The *GLR‐2* receptor–mediated glutamatergic signaling between ASH and AIA appears to function during the starvation period, while *GLC‐3* receptor–mediated signaling in AIA may play a role during the training phase. This circuit provides a molecular and neural mechanism by which the hunger state enhances subsequent food‐deprivation‐related aversive olfactory learning, underscoring the intricate neural pathways that govern learning and memory in response to environmental cues.

### 2.5 An Octopamine Neural Circuit Mediates Hunger‐Facilitated Appetitive Olfactory Learning

**Figure 5:**
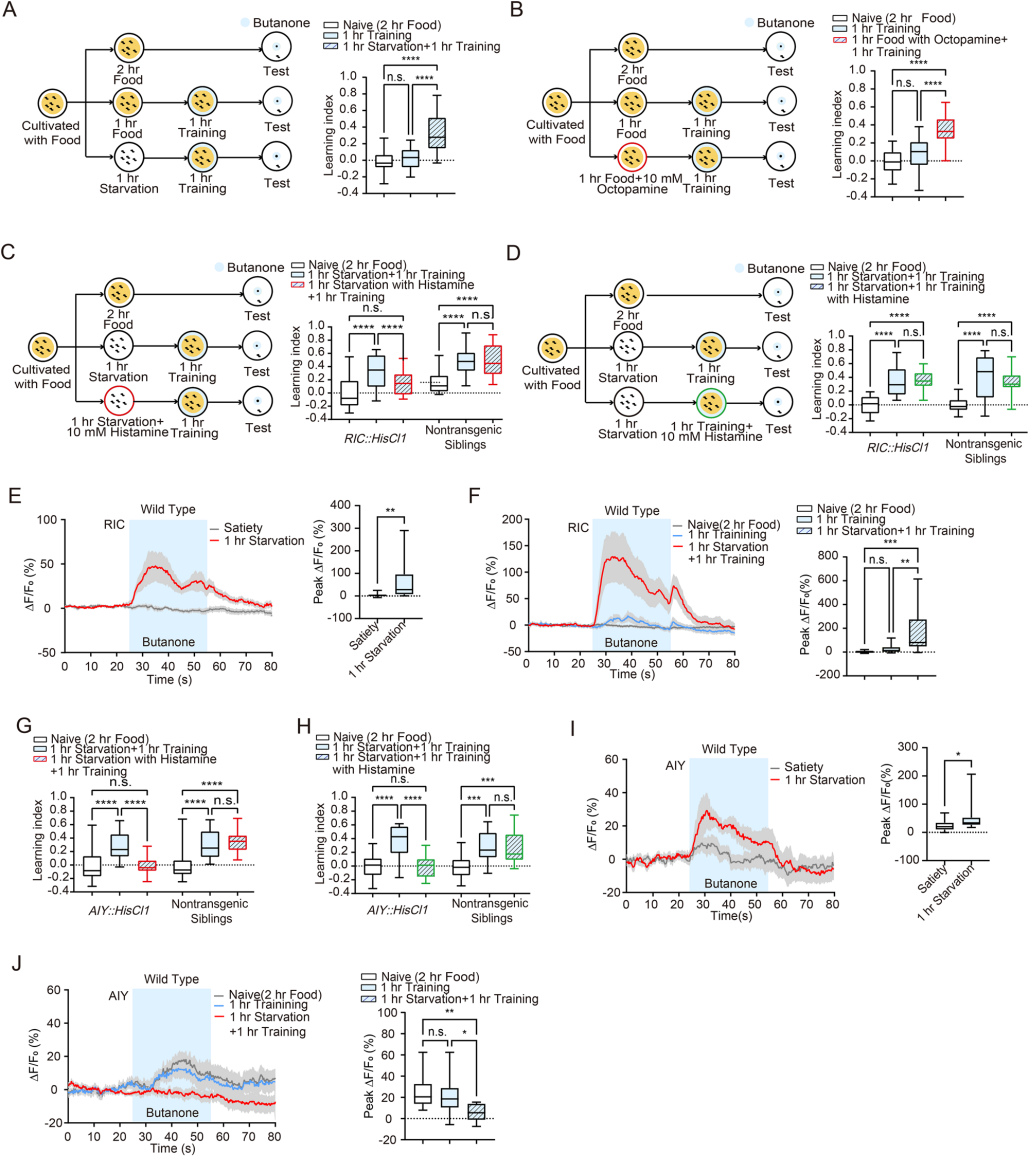
An octopamine neural circuit mediates hunger‐enhanced food‐presentation‐associated appetitive olfactory learning. A) Left: Schematic representation of the behavioral paradigm for appetitive olfactory learning. Right: Statistical comparison of the appetitive learning index among worms subjected to 1 h starvation followed by 1 h training (*n* = 21), naive animals (*n* = 21), and those subjected to 1 h training (*n* = 19). B) Schematic representation and statistical comparison of the behavioral paradigm for octopamine treatment during the 1 h starvation session (*n* = 20 per group). C) Schematic representation and statistical comparison of the behavioral paradigm for manipulating the RIC neuron with histamine treatment during the 1 h starvation session (*n* = 21 per group). D) Schematic representation and statistical comparison of the behavioral paradigm for manipulating the RIC neuron with histamine treatment during the 1 h training session (*RIC::HisCl1*: Naive, *n* = 20; 1 h Starvation+1 h Training, *n* = 20; 1 h Starvation+1 h Training with Histamine, *n* = 19; Nontransgenic Siblings: Naive, *n* = 19; 1 h Starvation+1 h Training, *n* = 20; 1 h Starvation+1 h Training with Histamine, *n* = 20). E) Left: Traces of *GCaMP6* signals in the RIC neuron in response to butanone under satiated and 1 h starvation conditions (*n* = 18 per group). Colored lines and light gray areas represent mean values and SEM, respectively. Right: Statistical comparison of peak *GCaMP6* signals in RIC during butanone exposure under satiated and 1 h starvation conditions. F) Left: Traces of *GCaMP6* signals in the RIC neuron in response to butanone under naive (*n* = 16), 1 h training (*n* = 15), and 1 h training followed by 1 h starvation conditions (*n* = 16). Colored lines and light gray areas represent mean values and SEM, respectively. Right: Statistical comparison of peak *GCaMP6* signals in RIC during butanone exposure under naive, 1 h training, and 1 h training followed by 1 h starvation conditions. G) Inhibition of AIY neuron during the 1 h starvation session abolishes hunger‐enhanced appetitive olfactory learning (*AIY::HisCl1*: *n* = 20 per group; Nontransgenic Siblings: *n* = 19 per group). (H) Inhibition of AIY neuron during the 1 h training session abolishes hunger‐enhanced appetitive olfactory learning (*AIY::HisCl1*: Naive, *n* = 18; 1 h Starvation+1 h Training, *n* = 18; 1 h Starvation+1 h Training with Histamine, *n* = 17; Nontransgenic Siblings: Naive, *n* = 17; 1 h Starvation+1 h Training, *n* = 18; 1 h Starvation+1 h Training with Histamine, *n* = 18). I) Left: Traces of *GCaMP6* signals in the AIY neuron in response to butanone under satiated and 1 h starvation conditions. Colored lines and light gray areas represent mean values and SEM, respectively. Right: Statistical comparison of peak *GCaMP6* signals in AIY during butanone exposure under satiated (*n* = 19) and 1 h starvation conditions (*n* = 17). J) Left: Traces of *GCaMP6* signals in the AIY neuron in response to butanone under naive, 1 h training, and 1 h training followed by 1 h starvation conditions. Colored lines and light gray areas represent mean values and SEM, respectively. Right: Statistical comparison of peak GCaMP6 signals in AIY during butanone exposure under naive (*n* = 12), 1 h training (*n* = 14), and 1 h training followed by 1 h starvation conditions (*n* = 13). Data are presented as boxplots showing the maximum value, 75th percentile, median, 25th percentile, and minimum value. Statistical significance was determined using Student's *t*‐test or two‐way ANOVA with Tukey's post hoc test. ^*^ *p* < 0.05; ^**^ *p* < 0.01; ^***^ *p* < 0.001; ^****^ *p* < 0.0001; n.s., not significant.

We have discovered that the hunger state enhances subsequent food‐deprivation‐associated aversive olfactory learning and elucidated the underlying neural circuit. We next investigated whether hunger also regulates food‐presentation‐associated appetitive olfactory learning. Indeed, previous studies have demonstrated that *C. elegans* exhibits an increased preference for butanone when it is associated with food following a period of starvation, although the underlying mechanisms remain poorly understood.^[^ ^30^, ^55^, ^56^, ^57^, ^58^ ^]^ To examine the influence of the hunger state on this appetitive learning, we introduced a starvation period before the appetitive training session. We found that a 1 h starvation significantly facilitated food‐presentation‐related appetitive olfactory learning, as evidenced by a significantly increased learning index under the 1 h starvation followed by 1 h training condition (**Figure** **5**A; Figure S9A, Supporting Information). These findings are consistent with previous studies.^[^ ^55^, ^56^ ^]^ To determine the role of octopamine signaling in this facilitation, we administered 100 µL of 10 mm octopamine to the worms with food for 1 h, then subjected them to a 1 h training session. The significantly increased learning index observed under this condition suggests that octopamine administration mimics the hunger state, even when the worms are physically satiated, thereby facilitating appetitive olfactory learning (Figure 5B; Figure S9B, Supporting Information).

Since the RIC neuron is the sole source of octopamine in *C. elegans*,^[^ ^32^ ^]^ we hypothesized that inhibiting RIC during the starvation period would attenuate hunger‐related appetitive olfactory learning facilitation. Consistent with this prediction, *RIC::HisCl1* transgenic worms treated with histamine during the 1 h starvation period exhibited a decreased learning index, a phenotype not observed in their nontransgenic siblings (Figure 5C; Figure S9C, Supporting Information). Notably, inhibiting RIC during the appetitive learning period did not affect hunger‐related appetitive learning facilitation (Figure 5D; Figure S9D, Supporting Information), indicating that the octopamine signal from the RIC neuron specifically functions during the starvation period. To further elucidate RIC's role in hunger‐related appetitive olfactory learning facilitation, we performed calcium imaging to examine RIC's calcium response to butanone. We found that RIC did not respond to butanone presentation under satiated conditions but displayed strong activation upon butanone stimulus after 1 h of starvation (Figure 5E). Furthermore, 1 h starvation followed by 1 h appetitive training induced significantly increased calcium events in RIC compared to naive and 1 h appetitive training alone conditions (Figure 5F), confirming RIC's essential role in hunger‐related appetitive olfactory learning facilitation.

Next, we sought to identify the downstream neuron of RIC involved in this process. Interneuron AIY was considered as a candidate because of its essential role in olfactory learning.^[^ ^51^ ^]^ We suppressed AIY activity during the 1 h starvation or 1 h appetitive learning period by administering histamine to *AIY::HisCl1* transgenic animals. *AIY::HisCl1* transgenic worms with histamine treatment during 1 h starvation or 1 h appetitive learning session exhibited a similar learning index to naive animals (Figure 5G,H; Figure S9E,F, Supporting Information), indicating that AIY is involved in both the starvation and learning periods to regulate hunger‐related appetitive learning facilitation. When examining AIY's response to butanone under different conditions, we found significant differences between the satiated and 1 h starved conditions (Figure 5I), as well as a significantly decreased calcium response to butanone after 1 h starvation followed by 1 h appetitive training (Figure 5J).

### 2.6 An Octopamine Receptor *SER‐6* Enhances the Appetitive Olfactory Learning

**Figure 6:**
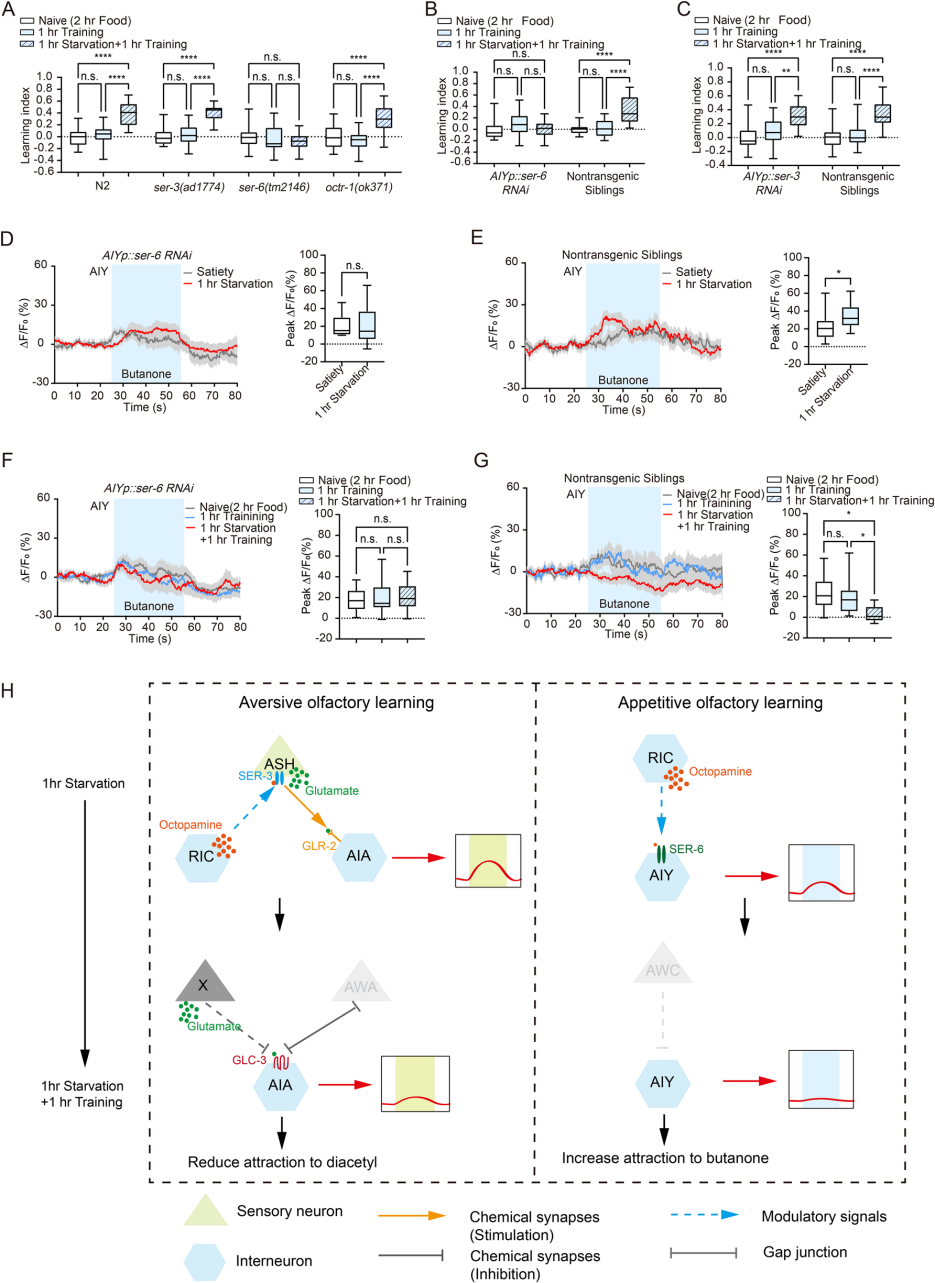
Octopamine receptor SER‐6 in AIY regulates hunger‐facilitated food‐presentation‐associated appetitive olfactory learning. A) Statistical comparison of the appetitive learning index among wild‐type (Naive: *n* = 21; 1 h Training: *n* = 20; 1 h Starvation + 1 h Training: *n* = 21), ser‐3 (*n* = 21 per group), ser‐6 (*n* = 21 per group), and octr‐1 (*n* = 20 per group) loss‐of‐function worms subjected to 1 h training followed by 1 h starvation, naive animals, and those subjected to 1 h training. B) Silencing ser‐6 expression in AIY abolishes hunger‐enhanced appetitive olfactory learning (*AIYp::ser‐6 RNAi*: Naive, *n* = 20; 1 h Training, *n* = 19; 1 h Starvation+1 h Training, *n* = 20; Nontransgenic Siblings: Naive, *n* = 18; 1 h Training, *n* = 21; 1 h Starvation+1 h Training, *n* = 21). C) Silencing ser‐3 expression in AIY does not affect hunger‐enhanced appetitive olfactory learning (*AIYp::ser‐3 RNAi*: Naive, *n* = 18; 1 h Training, *n* = 17; 1 h Starvation+1 h Training, *n* = 18; Nontransgenic Siblings: *n* = 18 per group). D,E) Silencing ser‐6 expression in AIY affects AIY's response to butanone odor after 1 h starvation (D: Satiety, *n* = 13; 1 h Starvation, *n* = 12; E: Satiety, *n* = 14; 1 h Starvation, *n* = 13). F,G) Silencing ser‐6 expression in AIY affects AIY's response to butanone odor after 1 h training followed by 1 h starvation (F: Naive, *n* = 12; 1 h Training, *n* = 10; 1 h Starvation+1 h Training, *n* = 10; G: Naive, *n* = 10; 1 h Training, *n* = 10; 1 h Starvation+1 h Training, *n* = 11). H) Working models of the circuitry and molecular mechanisms underlying hunger‐enhanced aversive and appetitive olfactory learning. Data are presented as boxplots showing the maximum value, 75th percentile, median, 25th percentile, and minimum value. Statistical significance was determined using Student's *t*‐test or two‐way ANOVA with Tukey's post hoc test. ^*^ *p* < 0.05; ^**^ *p* < 0.01; ^****^ *p* < 0.0001; n.s., not significant.

To confirm that AIY functions as a downstream neuron of RIC in regulating hunger‐related appetitive olfactory learning facilitation, we examined the role of three known octopamine receptors *SER‐3*, *SER‐6*, and *OCTR‐1*. We performed behavioral assays using loss‐of‐function mutants. Intriguingly, *ser‐6* mutants exhibited a similar learning index when trained 1 h following 1 h starvation (**Figure** **6**A; Figure S9G, Supporting Information), indicating *SER‐6* is involved in hunger‐related learning facilitation. To examine whether *SER‐6* on AIY conveys the octopamine signal from RIC to mediate this process, we knocked down *ser‐6* in AIY using a specific promoter and examined the learning behaviors. We found that *AIY::ser‐6 RNAi* transgenic animals did not exhibit hunger‐related appetitive olfactory learning facilitation (Figure 6B; Figure S9H, Supporting Information). However, knocking‐down *ser‐3* in AIY does not reveal this effect (Figure 6C; Figure S9I, Supporting Information). We further performed calcium imaging experiments to examine AIY's response to butanone in the *ser‐6* knockdown background. Results showed that *SER‐6* in AIY is involved in calcium dynamic changes during both 1 h starvation and 1 h starvation followed by 1 h appetitive learning (Figure 6D–G).

In summary, we identified a neural circuit, RIC→AIY, regulating hunger‐facilitated food‐presentation‐related appetitive olfactory learning. In this circuit, the octopaminergic neuron RIC participates during the starvation period, while the interneuron AIY is involved in both the starvation and appetitive learning periods (Figure 6H). This circuit provides a molecular and neural mechanism which the hunger state facilitates food‐presentation‐associated appetitive olfactory memory, emphasizing the neural circuit for cognitive flexibility responding to environmental changes.

### 2.7 Hunger State Facilitates Food‐Related Olfactory Learning in Rodents

We have elucidated that the state of hunger enhances both food‐deprivation‐associated aversive olfactory learning and food‐presentation‐associated appetitive olfactory learning in *C. elegans*, and have demonstrated the essential role of neuromodulator octopamine. To test whether this principle is conserved across species, we designed behavioral assays to assess food‐related olfactory learning in mice.

**Figure 7:**
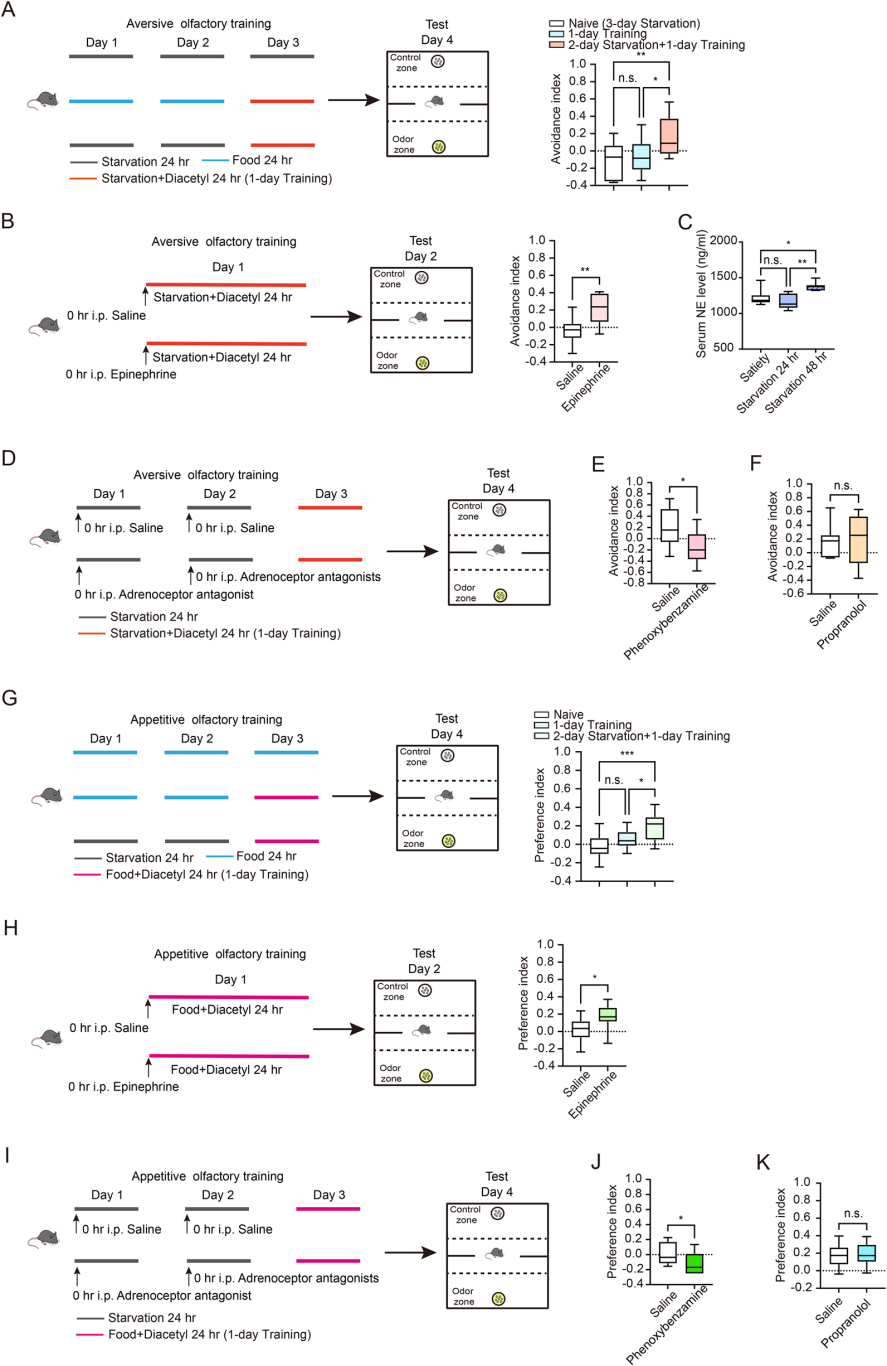
Norepinephrine signaling regulates hunger‐facilitated olfactory learning in mice. A) Schematic representation of the aversive olfactory learning paradigm in mice (Left) and statistical comparison of time spent in the empty chamber under naive (*n* = 15), 1‐day training (*n* = 12), and 1‐day training followed by 2‐day starvation conditions (*n* = 12) (Right). B) Schematic representation of epinephrine administration during the aversive olfactory learning (Left) and statistical comparison of time spent between epinephrine and saline administration (Right) (*n* = 9 per group). C) Statistical comparison of norepinephrine level in serum under satiety, 1‐day starvation, and 2‐day starvation conditions (*n* = 7 per group). D) Schematic representation of Phenoxybenzamine/Propranolol administration during the aversive olfactory learning. E) Statistical comparison of time spent between Phenoxybenzamine (*n* = 11) and saline (*n* = 12) administration groups after 1‐day training followed by 2‐day starvation conditions. F) Statistical comparison of time spent between Propranolol and saline administration groups after 1‐day training followed by 2‐day starvation conditions (*n* = 7 per group). G) Schematic representation of the appetitive olfactory learning paradigm in mice (Left) and statistical comparison of time spent in the empty chamber under naive (*n* = 16), 1‐day training (*n* = 16), and 1‐day training followed by 2‐day starvation conditions (*n* = 12) (Right). H) Schematic representation of epinephrine administration during the appetitive olfactory learning (Left) and statistical comparison of time spent between epinephrine (*n* = 13) and saline (*n* = 12) administration (Right). I) Schematic representation of Phenoxybenzamine/Propranolol administration during the appetitive olfactory learning. (J) Statistical comparison of time spent between Phenoxybenzamine (*n* = 14) and saline (*n* = 13) administration groups after 1‐day training followed by 2‐day starvation conditions. K) Statistical comparison of time spent between Propranolol (*n* = 10) and saline (*n* = 9) administration groups after 1‐day training followed by 2‐day starvation conditions. Data are presented as boxplots showing the maximum value, 75th percentile, median, 25th percentile, and minimum value. Statistical significance was determined using Student's *t*‐test or two‐way ANOVA with Tukey's post hoc test. ^*^ *p* < 0.05; ^**^ *p* < 0.01; ^****^ *p* < 0.0001; n.s., not significant.

To establish food‐deprivation‐associated aversive olfactory learning in mice, we introduced a filter mat infused with 4 mL of 1:10000 diluted diacetyl into their cages and deprived them of food for 24 h. Subsequently, we placed individual mice in a cage that contained three zones: one zone with diacetyl odor, one zone with H_2_O, and a middle zone for 11 min (**Figure** **7**A; Figure S10A, Supporting Information). The avoidance index of mice starved for 1 day followed by 1 day of training showed no significant increase compared with the naive group or those subjected to 1 day of training (Figure S10A, Supporting Information). In contrast, mice that were starved for 2 days and then trained to associate the diacetyl odor with food deprivation exhibited significantly greater avoidance of the diacetyl‐scented zone compared to the naive group and 1‐day training mice (Figure 7A). This suggests that prior starvation facilitates food‐deprivation‐related aversive olfactory learning in mice.

Previous studies have shown that intraperitoneal administration of epinephrine increases norepinephrine levels in the central nervous system via the vagal afferent–nucleus of the solitary tract (NTS) pathway.^[^ ^59^ ^]^ To further investigate the role of norepinephrine, an analog of vertebrate octopamine, in facilitating aversive learning, we administered an intraperitoneal dose of 1.5 mg kg^−1^ epinephrine at the start of the training session (Figure 7B). Mice treated with epinephrine exhibited significant avoidance of diacetyl‐scented zone (Figure 7B), indicating that norepinephrine administration mimics the hunger state that facilitates food‐deprivation‐related aversive olfactory learning. To determine whether peripheral norepinephrine influences subsequent olfactory learning, we first measured its levels in serum. Intriguingly, 2‐day starvation led to an increase in norepinephrine levels (Figure 7C). Next, we inhibited adrenergic receptor function to assess its effect on avoidance behavior. Alpha‐adrenergic and beta‐adrenergic receptors are the two major classes of norepinephrine receptors.^[^ ^60^, ^61^ ^]^ A previous study has shown that alpha‐1 receptors exhibit an excitatory effect,^[^ ^61^ ^]^ whereas beta receptors play an oppositive role in smooth muscle cells.^[^ ^62^ ^]^ Given that octopamine induces an excitatory effect under the hunger state in *C. elegans*, we hypothesized that alpha‐1 adrenergic receptor may be involved. To test this, we used the alpha‐1 adrenergic antagonist phenoxybenzamine, which blocks peripheral alpha‐adrenoceptor function,^[^ ^63^ ^]^ to assess its role in aversive olfactory learning (Figure 7D). The result displayed that blocking alpha1‐adrenergic receptors attenuated hunger‐facilitated aversive learning (Figure 7E). In contrast, inhibition of beta‐adrenergic receptors using propranolol (Figure 7D), which readily crosses the blood–brain barrier,^[^ ^64^ ^]^ had no effect on this learning process (Figure 7F).

To establish food‐presentation‐associated appetitive olfactory learning, we added 3 mL of 1:10000 diluted diacetyl to the cage for one day (Figure 7G). By examining zone preference after training, we found that a 1‐day association was insufficient to establish a preference for the diacetyl odor, whereas 2 days of starvation before appetitive learning produced a significant preference for diacetyl (Figure 7G). This indicates that prior hunger promotes subsequent food‐presentation‐related appetitive learning in mice. And this process seems to be mediated by norepinephrine, as evidenced by the significant preference toward diacetyl in mice with epinephrine treatment before appetitive learning (Figure 7H). Furthermore, administration of the alpha1‐adrenergic receptor antagonist phenoxybenzamine attenuated hunger‐facilitated appetitive learning, whereas the beta‐adrenergic antagonist propranolol had no effect (Figure 7I–K). These findings suggest a potential involvement of norepinephrine and the alpha1‐adrenergic receptor in hunger‐facilitated learning.

Overall, our findings indicate that hunger may facilitate food‐related olfactory learning in both *C. elegans* and mice, pointing to a possible cross‐species similarity in this process.

## 3 Conclusion and Discussion

Using well‐established aversive and appetitive learning paradigms in *C. elegans*,^[^ ^30^, ^55^, ^56^, ^58^, ^65^ ^]^ our study investigates the relationship between hunger and memory formation, with a focus on the neuromodulatory role of octopamine in hunger‐enhanced olfactory learning, rather than the fundamental mechanisms of learning itself. By examining both aversive and appetitive memory formation in *C. elegans* and mice, our study suggests the involvement of potentially conserved molecular mechanisms in hunger‐induced cognitive changes. These findings highlight the influence of physiological states on cognitive functions and provide insights into the molecular and neural pathways that may link hunger with memory processes.

### 3.1 Hunger Functions as a Cognitive Modulator

Hunger is a potent motivator that modulates a wide range of behaviors across species, influencing decision‐making, risk‐taking, and attention.^[^ ^66^, ^67^ ^]^ In humans, hunger has been shown to affect cognitive processes, enhancing the focus on food‐related stimuli while impairing tasks unrelated to food.^[^ ^9^, ^10^ ^]^ Studies in animals, such as rodents and insects, have shown that hunger can increase foraging activity and decrease the aversion to risk.^[^ ^68^, ^69^, ^70^ ^]^ Hunger also increases the salience of food‐related cues,^[^ ^71^, ^72^ ^]^ leading to improved recall of food‐associated information.^[^ ^73^, ^74^ ^]^ Our study emphasizes that hunger enhances both aversive and appetitive olfactory memory formation. Our study provides strong evidence that supports hunger as an important internal state that regulates cognitive behaviors.^[^ ^67^ ^]^

In this study, we demonstrate that 1 or 3 h starvation results in a distinct physiological state at the molecular level by analyzing the expression levels of 537 metabolites (Figure S1, Supporting Information). By considering satiety, 1 h starvation, and 3 h starvation as three distinct points along a continuous starvation process, we analyzed metabolic changes and found that the metabolites exhibited diverse dynamic patterns (Figure S1C–F, Supporting Information). Amino acids and their derivatives appear to be key factors in the physiological response to starvation. Among them, glutathione metabolism may influence the levels of the neurotransmitter glutamate, which in turn could regulate behavioral changes.^[^ ^6^ ^]^ Indeed, our study demonstrates that the glutamatergic sensory neuron ASH, along with two glutamate receptors, *GLR‐2* and *GLC‐3*, is involved in hunger‐facilitated learning. These findings suggest that, in addition to glutamate‐related pathways, other starvation‐responsive metabolites may also contribute to the modulation of behavior under nutrient‐deficient conditions.

At the circuitry level, the interneuron RIC responds to both the aversive odor diacetyl and the appetitive odor butanone after starvation, with calcium dynamics that differ significantly from those observed under satiated conditions. Thus, the RIC interneuron establishes a distinct brain state that facilitates subsequent learning at the behavioral level. Given its role in regulating various biological processes after starvation,^[^ ^19^, ^49^, ^75^ ^]^ it is reasonable to predict that RIC may respond to other stimuli as well. Sensory neuron AWA is the primary neuron responsible for diacetyl perception,^[^ ^76^, ^77^ ^]^ while sensory neuron AWC responds to butanone.^[^ ^55^ ^]^ However, neither of these neurons directly connects to RIC.^[^ ^42^, ^43^ ^]^ This raises several interesting questions. For instance, what neural circuit mediates the olfactory information flow towards RIC, and how this circuit is modified by starvation. It was observed that RIC exhibits an on response to odor stimuli, which is unlikely to result from shear stress (Figure S5A, Supporting Information), suggesting that RIC may receive complex sensory inputs following starvation.

The regulation of feeding is a complex and evolutionarily conserved behavior across the animal kingdom, shaped by both environmental cues and internal physiological states.^[^ ^78^, ^79^ ^]^ To maintain energy homeostasis and ensure the intake of essential nutrients, animals must continuously integrate external sensory information with internal metabolic signals. This integration is mediated by a diverse array of neurotransmitters and neuromodulators, including biogenic amines such as serotonin (5‐HT), dopamine, and norepinephrine in vertebrates, as well as tyramine and octopamine in invertebrates.^[^ ^80^, ^81^ ^]^ A previous study showed *C. elegans* relies on serotonin (5‐HT) signaling to exhibit food‐appropriate behavioral responses, whereas octopamine signaling mediates adaptations to fasting.^[^ ^20^ ^]^ Nutritional state modulates aversive responses in *C. elegans* through serotonergic signaling from two serotonergic neurons, NSM and ADF. NSM‐derived 5‐HT promotes aversive behavior by activating *SER‐5* receptors on ASH sensory neurons and *SER‐1* receptors on RIA interneurons. Conversely, ADF‐derived 5‐HT suppresses aversion by activating *SER‐1* on the octopaminergic RIC interneurons.^[^ ^82^ ^]^ Physiological studies have shown that, under nutrient‐rich conditions, serotonin released from ADF neurons inhibits octopamine release from RIC neurons. During starvation, this inhibitory effect is reduced, leading to increased octopamine release from RIC neurons, which in turn mediates starvation‐induced behavioral changes.^[^ ^21^ ^]^ These findings highlight the importance of investigating the role of 5‐HT in hunger‐facilitated learning.

### 3.2 Octopamine/Norepinephrine Signaling Regulates Hunger‐Enhanced Learning

Our findings underscore the critical role of octopamine in invertebrates and norepinephrine in vertebrates as key neuromodulators in hunger‐enhanced memory formation. We demonstrate that feeding worms octopamine mimics the hunger state, facilitating the formation of both aversive and appetitive memories, regardless of their physiological satiation. Similarly, norepinephrine administration enhances both aversive and appetitive learning in vertebrates. Mechanistically, octopamine signaling appears to be specifically involved during starvation, establishing the hunger state and modulating distinct downstream circuits for aversive and appetitive learning. Octopamine receptor *SER‐3* in ASH neuron is specifically involved in 1 h aversive learning followed by 1 h starvation, but not in regular 2 h aversive learning. These results provide evidence for the essential role of octopamine/norepinephrine signaling in the brain's hunger state. They also support the hypothesis that memory formed under satiated or starved conditions engages different neural circuits, despite producing similar behavioral outcomes. This highlights the importance of carefully considering experimental conditions when interpreting the underlying mechanisms of cognitive behaviors.^[^ ^83^, ^84^, ^85^ ^]^

Studies have demonstrated upstream regulators of octopamine/norepinephrine signaling in the context of hunger, particularly within mammalian nervous systems. For example, in *Drosophila*, adipokinetic hormone regulates the output of octopaminergic neurons to mediate shock‐ and bitter‐taste‐reinforced aversive learning.^[^ ^18^ ^]^ In *C. elegans*, octopamine and serotonin are two key modulators that represent feeding status and modify various behaviors.^[^ ^86^, ^87^, ^88^, ^89^ ^]^ The octopaminergic neuron RIC in *C. elegans* expresses a range of receptors, including dopamine and serotonin receptors.^[^ ^31^, ^90^, ^91^, ^92^, ^93^ ^]^ Thus, hunger‐induced learning facilitation in *C. elegans* or mice offers an excellent platform to investigate the regulatory interplay between different neuromodulators and how they modify behaviors in a hunger state.

Our findings also raise the possibility that the source of norepinephrine (NE) involved in hunger‐facilitated learning originates from the periphery rather than the central nervous system. Several observations support this hypothesis. First, we observed increased serum NE levels following 2 days of starvation (Figure 7C). Second, peripheral NE does not cross the blood–brain barrier, suggesting its actions are mediated outside the central nervous system (ref). Third, blocking peripheral alpha 1‐adrenoceptors with phenoxybenzamine, which does not cross the blood–brain barrier,^[^ ^63^ ^]^ attenuated the effect (Figure 7E,J), whereas propranolol, a beta‐adrenergic antagonist that does cross the barrier,^[^ ^64^ ^]^ did not affect hunger‐facilitated learning (Figure 7F,K). Together, these results point to a peripheral contribution of NE in regulating starvation‐induced behavioral modulation. Future studies will be needed to dissect the circuitry mechanisms by which peripheral NE signals are integrated into the neural processes underlying hunger‐facilitated learning.

According to this hypothesis, it is worth mentioning that the circuit level may be largely distinct, though with the conserved molecular level, when interpreting the function of octopamine and SER‐3/SER‐6 receptors and NE with alpha‐1 receptors,

### 3.3 Distinct Neural Circuits for Aversive and Appetitive Learning

A significant contribution of this study is the identification of distinct neural circuits underlying hunger‐enhanced aversive and appetitive learning. Previous studies have shown that AIA plays a key role in aversive learning.^[^ ^30^, ^39^, ^94^ ^]^ Here, we have discovered that the RIC → ASH → AIA circuit mediates the enhancement of aversive olfactory learning in *C. elegans*. In this circuit, octopamine signaling from the RIC neuron and glutamate signaling from the ASH neuron prime the system during starvation, while the AIA neuron is involved in both the starvation and learning phases. Our results confirmed that the AIA neuron regulates regular aversive olfactory learning.^[^ ^30^, ^39^ ^]^ However, the glutamate receptor *GLR‐2* in AIA is not involved in regular olfactory learning. This finding suggests that the integration of modulatory signals from ASH and learning signals from the sensory neuron AWA^[^ ^95^ ^]^ in AIA plays a key role during the training session in establishing aversive olfactory memory in the hunger state.

The ASH neuron was initially identified as a sensory neuron based on its morphology and physiological response to aversive cues.^[^ ^45^, ^46^, ^96^ ^]^ Consistently, we found that ASH activation after starvation, along with subsequent training, may mediate avoidance behaviors, thereby contributing to the formation of aversive memory. In the RIC → ASH → AIA circuit, ASH functions as an interneuron, modulating the activity of the AIA neuron after starvation. This supports the idea that individual neurons in the *C. elegans* neural network can serve multiple functions, allowing for diverse behavioral outputs.^[^ ^97^, ^98^ ^]^

Conversely, the circuit responsible for appetitive learning involves a different neural pathway, RIC → AIY, and the octopamine receptor *SER‐6*, reflecting the complexity and specificity of the mechanisms that govern distinct types of learning. Consistently, previous studies have shown that starvation modulates AIY activity via octopamine and *SER‐6* in CO_2_ sensory responses.^[^ ^99^ ^]^ These findings further support the idea that different types of memories are mediated by highly specialized neural substrates.^[^ ^100^ ^]^

Previous study has shown that AIY is involved in both appetitive and aversive learning related to butanone.^[^ ^58^ ^]^ Interestingly, both forms of learning lead to decreased calcium activity in response to butanone when it is presented following diacetyl as a background stimulus, although the extent of the reduction differs between these two learning forms.^[^ ^58^ ^]^ Our results support these findings, suggesting that reduced AIY activity may underlie appetitive learning for butanone. These findings may appear to conflict with the previous understanding that the AWC–AIY circuit mediates attractive behaviors toward isoamyl alcohol (IAA),^[^ ^101^ ^]^ given that AWC is the primary sensory neuron for both isoamyl alcohol and butanone,^[^ ^102^, ^103^ ^]^ and AIY is the main postsynaptic partner of AWC.^[^ ^42^, ^43^ ^]^ However, some studies have reported that AWC responses to butanone are reduced after aversive learning,^[^ ^104^, ^105^ ^]^ which appears to conflict with AWC's established role in isoamyl alcohol (IAA) attraction, where decreased AWC activity is associated with enhanced attraction to IAA.^[^ ^101^, ^106^ ^]^ Above all, it suggests a more nuanced role for AWC‐AIY circuit in odor‐driven chemotaxis and behavioral plasticity.

### 3.4 The Navigation Index as an Indicator of Learning Effect

Luo et al. defined thermotaxis efficiency as the ratio of the mean velocity in the gradient direction to the overall crawling speed for each trajectory, in order to evaluate worms’ strategies in a linear temperature gradient.^[^ ^107^ ^]^ We adapted this method to assess navigational strategies as well as the learning effect during chemotaxis in a radial odor gradient.^[^ ^5^, ^6^ ^]^ As a ratio of two directional components, this parameter is not affected by the worm's speed or other locomotive features. Indeed, we have shown that 1 h starvation followed by 1 h training does not affect the worm's speed, however, it does induce aversive learning toward diacetyl (Figure S2, Supporting Information). We measured the speed of the worm strains in the absence of OP50, including mutants and transgenic animals (Figures S2,S4,S11–S13, Supporting Information). Although the absolute locomotion speeds we observed vary across experimental sets and can be higher than those reported in some previous studies,^[^ ^108^, ^109^ ^]^ our conclusions are robust because all genotype comparisons were performed under identical conditions within each set of experiments.

### 3.5 Comparative Analysis Across Species

We found that 1 h of training in *C. elegans* and 1 day of training in mice were insufficient to elicit a measurable learning effect. However, introducing a sufficiently long starvation period facilitates learning at these time points (Figures 1A,5A and 7A,G). Despite the differences in time scales, the resulting behavioral outcomes were remarkably similar, suggesting that conserved mechanisms may underlie associative learning across phyla. The parallels between *C. elegans* and mammalian systems in hunger‐enhanced memory formation suggest a conserved evolutionary strategy for optimizing cognitive function under conditions of nutrient scarcity. While the specific neural circuits and molecular players may differ, the overarching role of octopamine/norepinephrine signaling in linking hunger with memory appears to be a common theme across diverse species. This comparative approach not only broadens our understanding of the basic mechanisms of learning and memory but also provides a framework for exploring how these processes may be conserved or adapted in other organisms.

Our results highlight both conservation and divergence in the receptor mechanisms underlying hunger‐facilitated learning across species. In *C. elegans*, the octopamine receptors *SER‐3* and *SER‐6* are expressed in neurons and are required for starvation‐dependent learning. In mice, however, we find that alpha 1‐adrenergic receptors mediate this effect, and pharmacological evidence indicates that these receptors act in the periphery rather than in the central nervous system. One possible explanation is evolutionary specialization: invertebrate octopamine receptors display mixed features of mammalian alpha and beta‐adrenergic receptors, and *SER‐3/SER‐6* signaling may represent an ancestral neural mechanism. In mammals, adrenergic receptor subtypes have expanded and differentiated, allowing peripheral alpha 1 signaling to function as a systemic indicator of metabolic state that can modulate neural plasticity indirectly. Thus, while receptor subtype usage differs, the conserved theme is that adrenergic/octopaminergic pathways bridge starvation signals and learning circuits.

In conclusion, this study provides compelling evidence that hunger enhances both aversive and appetitive olfactory learning through distinct neural circuits, mediated by conserved octopamine/norepinephrine signaling. These findings contribute to a deeper understanding of the complex interplay between physiological states and cognitive function, revealing how the brain adapts to environmental challenges to optimize survival.

## 4 Experimental Section

### Elegans Strains

*C. elegans* strains were maintained at 20 °C according to the protocol outlined in WormBook.^[^ ^110^ ^]^ To generate transgenic lines with extrachromosomal arrays, the microinjection mixtures typically contained a total of 100 ng µL^−1^ plasmids. Each plasmid listed below was used at a concentration around 30 ng µL^−1^ in the mixture. If the total plasmid concentration was below 100 ng µL^−1^, pUC19 was added to bring the mixture to 100 ng µL^−1^. Integration line was generated as previously described^[^ ^111^ ^]^ with a microinjection setup (Xenoworks Digital Microinjector, Sutter Instrument). The following strains were used in this study (Table 1):

**Table 1:** Strains used in this study.

Strain	Genotype
N2 Bristol	*Wild‐type*
MT9971	nls107*[tbh‐1::GFP + lin‐15(+)]*
MT9455	*tbh‐1(n3247) X*
CX13079	*octr‐1(ok371) X*
CX12800	*ser‐3(ad1774) I*
FX17797	*ser‐6(tm2146) IV*
PSC339	*scnEx261[sra‐6p::HisCl; CC::GFP]*
PSC361	*scnEx283[tbh‐1p::HisCl; CC::GFP; lin‐44p::GFP]*
PSC248	*scnEx187[gcy‐28d::stop::HisCl; CC::GFP; cho‐1p::flp]*
PSC355	*scnEx277[tbh‐1p::GCaMP6s; lin‐44p::GFP; CC::DsRed]*
PSC6	*scnIs1[sra‐6p::GCaMP6s::F2A::mCherry; CC::DsRed]*
PSC265	*scnEx204[cho‐1p::flp; gcy‐28d::stop::GCaMP6s; CC::DsRed]*
PSC359	*scnEx281[sra‐6p::ser‐3 sense; sra‐6p::ser‐3 anti‐sense; CC::GFP]*
PSC449	*scnEx323[cho‐1p::glr‐2 sense; gcy‐28dp::glr‐2 anti‐sense; CC::GFP]*
PSC458	*scnEx330[sra‐6p::eat‐4 sense; sra‐6p::eat‐4 anti‐sense; CC::GFP]*
PSC282	*scnEx219[ttx‐3pG::HisCl; CC::GFP]*
PSC158	*scnEx110[ttx‐3pG::GCaMP6s; CC::DsRed]*
PSC524	*scnEx358[ttx‐3pG::ser‐6 sense; ttx‐3pG::ser‐6 anti‐sense; CC::GFP]*
BNU088	*zhuEx076[cho‐1p::glc‐3 sense; gcy‐28dp::glc‐3 anti‐sense; CC::GFP]*
BNU029	*zhuEx020[ttx‐3pG::ser‐3 sense; ttx‐3pG::ser‐3 anti‐sense; CC::GFP]*
BNU007	*scnEx281;scnls1 [mated by PSC359 and PSC6]*
BNU008	*scnEx323;scnEx204 [mated by PSC449 and PSC265]*
BNU009	*scnEx358;scnEx110 [mated by PSC524 and PSC158]*
BNU101	*zhuEx076;scnEx110 [mated by BNU088 and PSC158]*

### Molecular Cloning of Plasmids

The plasmids used in this study were generated by Gibson assembly,^[^ ^112^ ^]^ or Gateway Recombinational Cloning (Table S2, Supporting Information),^[^ ^113^ ^]^ or traditional ligation based on L4 ligase, following the manufacturer's protocol (Gibson Assembly Master Mix, NEB, E2611; Gateway LR Clonase II Enzyme mix, Gateway, 11791020). DNA fragments with overlapping arms for Gibson assembly were generated via PCR (2× High‐Fidelity PCR Master Mix, MedChemExpress, HY‐K0533, A300 Fast Thermal Cycler, Hangzhou LongGene Scientific Instruments Co., Ltd.). The primer, PCR template, and DNA fragment details are listed in Table S2 (Supporting Information). All plasmids were verified by sequencing. For expression in ASH, RIC, and AIY neurons, the 3269 bp *sra‐6* promoter,^[^ ^114^ ^]^ 2819 bp *tbh‐1* promoter,^[^ ^49^ ^]^ and 834 bp *ttx‐3* promoter G^[^ ^115^ ^]^ were used, respectively. AIA‐specific expression was achieved using an overlapping strategy with the 2875 bp *gcy‐28d* promoter^[^ ^116^ ^]^ and the 347 bp *cho‐1* promoter.^[^ ^117^ ^]^ Detailed sequence information for these promoters is provided in Table S2 (Supporting Information).

### Cell‐Specific Gene Knock‐Down

Cell‐specific gene knockdown was carried out as reported previously,^[^ ^118^ ^]^ with some modifications. Basically, double‐stranded RNAs, consisting of both sense and antisense RNA, were transcribed using cell‐specific promoters, e.g. *sra‐6* promoter for ASH neurons, *ttx‐3* promoter G for AIY neurons, and *gcy‐28d* and *cho‐1* promoter for their overlapping in AIA neurons. The DNA fragments responsible for generating these double‐stranded RNAs were subcloned into a plasmid using Gibson assembly. All these plasmids were verified by sequencing. Plasmid details can be found in Table S2 (Supporting Information). These plasmids, along with a microinjection marker plasmid (CC::GFP), were then injected into the gonad of *C. elegans* to create RNAi transgenic lines, specifically PSC359, PSC449, PSC458, PSC524, and BNU029.

### Sample Preparation for LC‐MS/MS

Samples were prepared for LC‐MS/MS analysis following a modified version of a previously described protocol.^[^ ^119^ ^]^ Briefly, samples were thawed completely, and 50 mg (± 2.5 mg) was weighed into a centrifuge tube. To each sample, 1 mL of 80% methanol (pre‐chilled to −20 °C) was added, and the samples were centrifuged at 2500 rpm for 2 min. The samples were then subjected to three freeze–thaw cycles: freezing in liquid nitrogen for 5 min, thawing on ice for 5 min, followed by vortexing for 2 min. After the final cycle, the samples were centrifuged at 12 000 rpm for 10 min at 4 °C. A 300 µL aliquot of the supernatant was transferred to a new centrifuge tube, frozen at −20 °C for 30 min, and centrifuged again at 12,000 rpm for 10 min at 4 °C. Finally, 200 µL of the supernatant was passed through a protein precipitation plate and stored at −20 °C until analysis. Nine samples were collected and divided into three groups: Well‐fed, 1 h starvation, and 3 h starvation.

### LC‐MS/MS Analysis

LC‐MS/MS analysis was performed using an Ultra Performance Liquid Chromatography (UPLC) system coupled with Tandem Mass Spectrometry (MS/MS). Methanolic extracts prepared as described above were injected onto a Waters ACQUITY UPLC HSS T3 C18 column (100 mm × 2.1 mm, 1.7 µm particle size) maintained at 40 °C, with a flow rate of 0.35 mL min^−1^. The mobile phase consisted of 0.05% formic acid in water (Solvent A) and 0.05% formic acid in acetonitrile (Solvent B). The gradient elution profile was as follows: 0–7 min, 95% A to 5% B; 8–9.5 min, 5% A to 95% B; and 9.6–12 min, 95% A to 5% B. The injection volume was set to 2 µL for all analyses.

Mass spectrometry was conducted under the following conditions: electrospray ionization temperature of 550 °C, spray voltage of 5.5 kV for positive mode and −4.5 kV for negative mode, and curtain gas pressure of 35 psi. Each ion pair was scanned using optimized declustering potential and collision energy settings.

### Aversive Olfactory Learning Paradigm in *C. elegans*

The aversive olfactory learning paradigm in *C. elegans* was modified according to the previous study.^[^ ^104^ ^]^ The training plate was prepared by evenly spreading 100 µL of 1:10000 diluted diacetyl (Shanghai Macklin Biochemical Technology Co., Ltd.) on the surface of a 6 cm NGM plate, which was then allowed to dry. A control plate was prepared by spreading 100 µL of ddH2O on the surface of an NGM plate and drying it under the same conditions, serving as the naive control. To initiate the training session, 20–30 one‐day‐old adult hermaphrodites were transferred to either the training plate or the naive control plate. In the starvation followed by training group, 20–30 one‐day‐old adult hermaphrodites were first starved for 1 h on an empty NGM plate and then transferred to the training plate for 1 h.

### Appetitive Olfactory Learning Paradigm in *C. elegans*

The appetitive olfactory learning paradigm in *C. elegans* was modified as previously described.^[^ ^55^, ^57^ ^]^ The training plates were prepared by evenly spreading 200 µL of OP50 (a strain of *Escherichia coli*) culture mixed with 100 µL of 1:10000 diluted butanone (Sigma‐Aldrich Ltd.) and allowing the plates to dry. A plate containing 200 µL of OP50 culture without butanone served as the naïve control. For the training procedure, 20–30 one‐day‐old adult hermaphrodites were placed on either the training plate or the control plate. Additionally, 20–30 one‐day‐old adult hermaphrodites were first starved for 1 h on an empty NGM plate before being transferred to the training plate for 1 h; these worms constituted the starvation followed by training group.

### Exogenous Octopamine Administration

OP50 culture (200 µL) was mixed with 100 µL of 10 mm octopamine solution (Sigma‐Aldrich Ltd.) and evenly spread on the surface of a 6 cm plate. After drying for about 1 h, 20–30 one‐day‐old adult worms were placed on the plate for 1 h to elevate their octopamine levels.

### Neural Activity Manipulation With Histamine Treatment

To inhibit individual neurons during the starvation period, an empty NGM plate was prepared by evenly spreading 100 µL of 10 mm histamine‐dihydrochloride (H7125, Sigma‐Aldrich) on its surface. For neuron inhibition during the training session, 100 µL of 10 mm histamine‐dihydrochloride (H7125, Sigma‐Aldrich) was similarly added to the surface of the training plate. After allowing the plate to dry for xx hours, 20–30 one‐day‐old transgenic animals expressing HisCl1 in specific neurons were placed on the plate for 1 h.

### Chemotactic Steering Assay

The chemotactic steering assay was conducted following the protocols established in previous studies.^[^ ^5^, ^6^ ^]^ A 10 µL droplet of 1:10000 diluted diacetyl (Shanghai Macklin Biochemical Technology Co., Ltd.) or 1:100000 diluted butanone (Sigma‐Aldrich Ltd.) was placed in the center of a 9 cm NGM plate. A one‐day‐old adult was then positioned 1.5 cm away from the odor droplet. The entire process, from placing the worm on the plate to reaching the edge of the odor drop, or up to a total of 5 min if the worm did not reach the drop, was recorded. The trajectory of the animal was recorded at 10 frames per second using a high‐speed camera (MV‐CA060‐11GM, Hikvision). The Navigation Index for each trajectory was calculated using custom‐built Nematode Trajectory Analysis software. Briefly, each worm was treated as a point defined by its center, and the sampling interval was 2 s. At each position *i*, the Navigation Index was defined as the cosine of the angle between the vector from position *i* to *i+1* and the vector from position *i* to the trajectory endpoint. The average of these values across the trajectory was taken as the Navigation Index for the individual worm. The Navigation Index, ranging from −1 to 1, indicates the attractiveness of the odor, with −1 representing strong aversion and 1 representing strong attraction. To ensure a positive value for the learning index, the learning index for aversive olfactory learning was calculated following the formula below: Learning index (i) = mean (Navigation index (naive)) − Navigation index (i)

(1) $$\begin{array}{ccc} \mathrm{Learning}\;\mathrm{index}\;\left(i\right) & = & \;\mathrm{mean}\;\left(\mathrm{Navigation}\;\mathrm{index}\;\left(\mathrm{naive}\right)\right)\;\\ & & -\;\mathrm{Navigation}\;\mathrm{index}\;\left(i\right) \end{array}$$

The learning index for appetitive olfactory learning was calculated following the formula below: Learning index (i) = Navigation index (i) − mean (Navigation index (naive))

(2) $$\begin{array}{ccc} \mathrm{Learning}\;\mathrm{index}\;\;\left(i\right) & = & \;\mathrm{Navigation}\;\mathrm{index}\;\left(i\right)\\ & & -\;\mathrm{mean}\;\left(\mathrm{Navigation}\;\mathrm{index}\;\left(\mathrm{naive}\right)\right) \end{array}$$

### Calculation of Locomotion Speed in *C. Elegans*

The chemotaxis behavior of *C. elegans* was recorded at a frame rate of 10 frames per second using a MV‐CA060‐11GM camera (Hikvision) to track the movement of the worm's centroid over time. The worm's average speed was calculated by dividing the total travel distance by the total time.

### In Vivo Calcium Imaging With Microfluidic System

A custom‐built microfluidic system was established based on principles from a previous study.^[^ ^33^ ^]^ Electrical valves, controlled by custom‐made software, regulated the liquid flow within the microfluidic channels made of PDMS. Individual one‐day‐old adult worms were immobilized in the microfluidic system, positioned above a 60 X oil‐immersion objective on a confocal Nikon Eclipse Ti2‐E inverted microscope equipped with an A1R spectral detector. Images were acquired at a frame rate of 4 frames per second. The odor stimulation protocol was as follows: 60 s of NGM buffer (A total of 1 L solution was prepared containing: 3.0 g NaCl, 975 mL ddH_2_O, 25 mL potassium phosphate buffer (pH 6.0), 1.0 mL CaCl_2_, and 1.0 mL MgSO_4_), followed by 30 s of odor exposure, and then 30 s of NGM buffer. Images were analyzed using ImageJ software, with the TurboReg plugin employed to align the raw images. Regions of interest (ROIs) were manually drawn around the somas. The average intensity of individual ROIs was obtained in ImageJ, with the mean intensity of 100 frames prior to odor stimulation defined as F_0_. ΔF was calculated as ΔF = F – F_0_, where F represents the fluorescence intensity of the ROI in each frame. The average value over a 2 s window centered around the maximum ΔF/F_0_ was defined as the peak ΔF/F_0_ for subsequent statistical analysis.

### Whole‐Cell Patch Clamp on RIC Neuron

MT9971 strain specifically labels RIC by expressing GFP in the RIC neuron.^[^ ^120^ ^]^ Electrophysiological recordings were performed on the GFP‐labeled RIC neuron inside the young adult hermaphrodites (strain MT9971) at room temperature as previously described.^[^ ^121^ ^]^ Worms of the satiated group were directly picked off plates seeded with an abundance of OP50. Starved worms were picked from the same seeded plates, washed with DPBS, and then placed on an unseeded NGM plate for 1–6 h before dissection. The gluing and dissection were performed under an Olympus SZX16 stereomicroscope equipped with a 1X Plan Apochromat objective and widefield 10X eyepieces. Briefly, an adult animal was immobilized on a Sylgard‐coated (Sylgard 184, Dow Corning Inc.) glass coverslip in a small drop of DPBS (D8537, Sigma‐Aldrich Ltd.) by applying a cyanoacrylate adhesive (Vetbond tissue adhesive, 3 m Company) along one side of the body. A puncture in the cuticle away from the incision site was made to relieve hydrostatic pressure. A small longitudinal incision was then made using a diamond dissecting blade (Type M‐DL 72029 L, Electron Microscopy Sciences) along the glue line adjacent to the neuron of interest. The cuticle flap was folded back and glued to the coverslip with GLUture Topical Adhesive (Abbott Laboratories), exposing the neuron to be recorded. The coverslip with the dissected preparation was then placed into a custom‐made open recording chamber (≈1.5 mL volume) and treated with 0.5 mg mL^−1^ collagenase (type IV, Sigma‐Aldrich Ltd.) for ≈10 s by hand pipetting. The recording chamber was subsequently perfused with the standard extracellular solution using a custom‐made gravity‐feed perfusion system for ≈10 mL.

All electrophysiological recordings were performed with the bath at room temperature under an upright microscope (Axio Examiner, Carl Zeiss Inc.) equipped with a 40X water immersion lens and 16X eyepieces. RIC neurons were identified by fluorescent markers and their anatomical positions. Preparations were then switched to the differential interference contrast (DIC) setting for patch‐clamp. Electrodes with resistance (RE) of 15–25 MΩ were made from borosilicate glass pipettes (BF100‐58‐10, Sutter Instruments) using a laser pipette puller (P‐2000, Sutter Instruments) and fire‐polished with a microforge (MF‐830, Narishige International USA Inc.). The standard pipette solution was (all concentrations in mM): [K‐gluconate 115; KCl 15; KOH 10; MgCl2 5; CaCl2 0.1; Na2ATP 5; NaGTP 0.5; Na‐cGMP 0.5; cAMP 0.5; BAPTA 1; Hepes 10; Sucrose 50], with pH adjusted with KOH to 7.2, osmolarity 320–330 mOsm. The standard extracellular solution was: [NaCl 140; NaOH 5; KCL 5; CaCl2 2; MgCl2 5; Sucrose 15; Hepes 15; Dextrose 25], with pH adjusted with NaOH to 7.3, osmolarity 330–340 mOsm. Liquid junction potentials were calculated and corrected before recording. Whole‐cell current clamp and voltage clamp experiments were conducted on an EPC‐10 amplifier (EPC‐10 USB, HEKA Elektronik) using PatchMaster software (HEKA Elektronik). Two‐component capacitive compensation was optimized at rest, and the series resistance was compensated to 50%. Analog data were filtered at 2 kHz and digitized at 10 kHz.

### Aversive Olfactory Learning Paradigm in Mice

A filter mat was placed to fully cover the bottom of a rodent home cage. During the training session, 2 mL of a 1:10000 dilution of diacetyl (Shanghai Macklin Biochemical Technology Co., Ltd.) was randomly applied to the filter mat. Two 8‐week‐old male C57BL/6 mice were then introduced into the cage and kept for 24 h with access to water but no food. After 12 h, an additional 1 mL of the 1:10000 diacetyl solution was added to the filter mat to maintain the odor within the cage. In the control group, two mice were housed in an identical cage for 24 h without exposure to the diacetyl odor.

### Appetitive Olfactory Learning Paradigm in Mice

To form an association between diacetyl odor and food supply, two 8‐week‐old male C57BL/6 mice were housed for 24 h in a standard rodent cage with 2 mL of 1:10000 diluted diacetyl randomly applied to sterilized wood shavings in the cage.

### Drugs Administration for Mice During Training

For the aversive olfactory training, epinephrine (Sigma‐Aldrich Ltd.) was dissolved in saline solution and administered intraperitoneally at a dose of 1 mg kg^−1^ at the beginning of the training session, with the same dose administered again 12 h later. For the appetitive learning, epinephrine was administered IP at a dose of 1.5 mg kg^−1^.

For the starvation session, mice were intraperitoneally injected with either saline, phenoxybenzamine (20 mg kg^−1^; Shanghai Macklin Biochemical Technology Co., Ltd.), or propranolol (20 mg kg^−1^; Shanghai Macklin Biochemical Technology Co., Ltd.) on Day 1. Following the injection, the mice were placed in an empty cage with access to water only. The same procedure was repeated on the following day.

### Diacetyl Odor Preference Assay

After training, individual mice were introduced into a cage containing two 45 cm‐long chambers. In one of the chambers, a cotton ball saturated with 200 µL of 1:10000 diluted diacetyl was placed. The trajectory of the mice was recorded for 11 min using an IR123GM‐60 camera (Fairsion Tech Ltd.). Trajectories from 2nd min to 11th min were analyzed, and the time spent in each chamber was calculated using Tracking Master V5 (VST GmbH).

### Collection and Analysis of Mouse Serum Norepinephrine Levels

Blood samples were collected from mice under three different conditions: well‐fed (satiety), 1‐day starvation, and 2‐day starvation. During blood collection, mice were anesthetized with isoflurane. Blood was drawn by inserting a glass capillary tube into the medial canthus of the eye. The collected blood samples were left to stand at room temperature for 30 min, then centrifuged at 3000 rpm and 4 °C for 20 min. The supernatant was carefully collected and transferred into pre‐labeled storage tubes, which were stored at −80 °C until further analysis. Norepinephrine levels in the serum were measured using a high‐sensitivity norepinephrine ELISA kit following the manufacturer's instructions (JLC3100/96, Shanghai Jingkang Bioengineering Co., Ltd.).

### Statistical Analysis

Statistical analysis and data visualization were performed using GraphPad Prism (version 9.5). The statistical tests included unpaired t‐tests and one‐way ANOVA. All statistical analyses were conducted following protocols in GraphPad Prism software. Asterisks indicate significant differences (^****^ *p* < 0.0001, ^***^ *p* < 0.001, ^**^ *p* < 0.01, and ^*^ *p* < 0.05).

### Ethics Statement

All the experimental procedures were performed under the Guide for the Care and Use of Laboratory Animals: Eighth Edition (ISBN‐10: 0‐309‐15396‐4). We have complied with all relevant ethical regulations for animal testing, and all animal studies were approved by the Animal Ethics Committee, Beijing Normal University (Permission ID:AECBNUZ2025018).

## Conflict of Interest

The authors declare no conflict of interest.

## Supporting information



Supporting Information

Supporting Information

Supporting Information

## Data Availability

All data generated or analyzed in the Manuscript and Supplementary information are listed in the Source data file. Any additional information required to reanalyze the data is available from the corresponding authors upon request. Source data are provided with this paper.
