# Supplementary material for: Hungry for Knowledge: Octopamine Signaling Regulates Hunger‐Enhanced Olfactory Learning
Source: Adv Sci (Weinh). 2025 Dec 15;13(12):e13842. doi: 10.1002/advs.202513842 (PMC12948213; doi:10.1002/advs.202513842)
Supplement: Supplementary file 1 — Supporting Information [file ADVS-13-e13842-s003.docx]

***Supporting Information***

**Hungry for Knowledge: Octopamine Signaling Regulates Hunger-Enhanced Olfactory Learning**

*Huijuan Zhao ^1,2,3,#^, Guiyuan Shi ^1,2,3,#^, Ruixue Qin ^1,2,3^, Yinghao Sun ^1,2,3^, Wenbo Guo ^5^, Ruixia Shi ^1^, Minxian Peng ^6^, Jingxuan Yang ^4^, Jianjian Zhao ^7^, Qiuhan Liu ^1^, Jun Xiao ^8^, Ke Zhang ^1,2,3^, Qiang Liu ^6^, Wenxing Yang ^4,*^, He Liu ^1,2,3,*^*

1. Department of Systems Science, Faculty of Arts and Sciences, Beijing Normal University, Zhuhai, Guangdong, China.
2. International Academic Center of Complex Systems, Beijing Normal University, Zhuhai, Guangdong, China.
3. School of Systems Science, Beijing Normal University, Beijing, China.
4. Department of Physiology, West China School of Basic Medical Sciences & Forensic Medicine, Sichuan University, Chengdu, Sichuan, China
5. MOE Key Lab of Bioinformatics, BNRIST Bioinformatics Division, Department of Automation, Tsinghua University, Beijing, China
6. Department of Neuroscience, City University of Hong Kong, Tat Chee Avenue, Kowloon Tong, Hong Kong, China.
7. Current address: Guangdong Institute of Intelligence Science and Technology, Zhuhai, Guangdong, China.
8. Current address: Laboratory Safety and Equipment Management Office, Beijing Normal University, Zhuhai, Guangdong, China.

# These authors contributed equally to this work.

* Corresponding author (s) E-mail (s): heliu@bnu.edu.cn; yangwx@scu.edu.cn


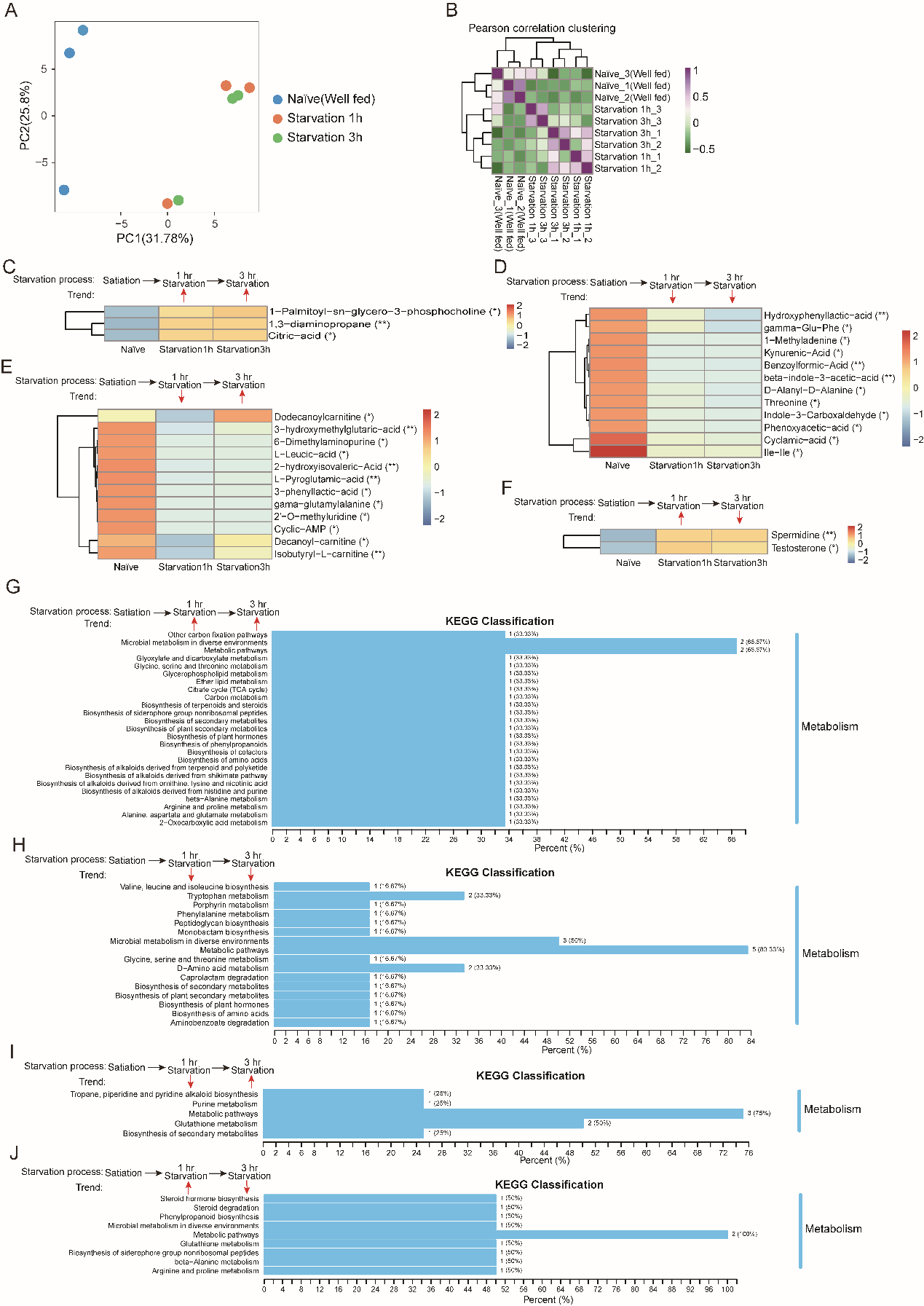


**Supplementary Figure 1. The levels of metabolites under satiated, 1-hour starvation, and 3-hour starvation conditions in C. elegans.**

1. Plot of the first two principal components (PC1 vs. PC2) illustrating the separation of different experimental groups. Each point represents an individual sample, with color coding indicating the different conditions. (B) Heatmap representing Pearson correlation coefficients between different experimental groups. The color scale indicates the strength and direction of the correlation, with purple representing strong positive correlations and green representing strong negative correlations. (C) Heatmap representing metabolites that follow a pattern of decrease followed by an increase, when considering satiation, 1-hour starvation, and 3-hour starvation as a continuous starvation process. Only metabolites with an adjusted p-value (after Benjamini-Hochberg correction) below 0.05 are presented. The statistical significances are labeled on the metabolite names (* denotes p-value < 0.05 and ** denotes p-value < 0.01). The following subfigures (D-F) utilize the same criterion. (D) Heatmap representing metabolites that follow a pattern of increase followed by a decrease, when considering satiation, 1-hour starvation, and 3-hour starvation as a continuous starvation process. (E) Heatmap representing metabolites that show a trend of continuous decrease across satiation, 1-hour starvation, and 3-hour starvation as a continuous starvation process. (F) Heatmap representing metabolites that show a trend of continuous increase across satiation, 1-hour starvation, and 3-hour starvation as a continuous starvation process. (G-J) KEGG classification of metabolites shown in panels (C–F), with a particular emphasis on those associated with the "Metabolism" category.


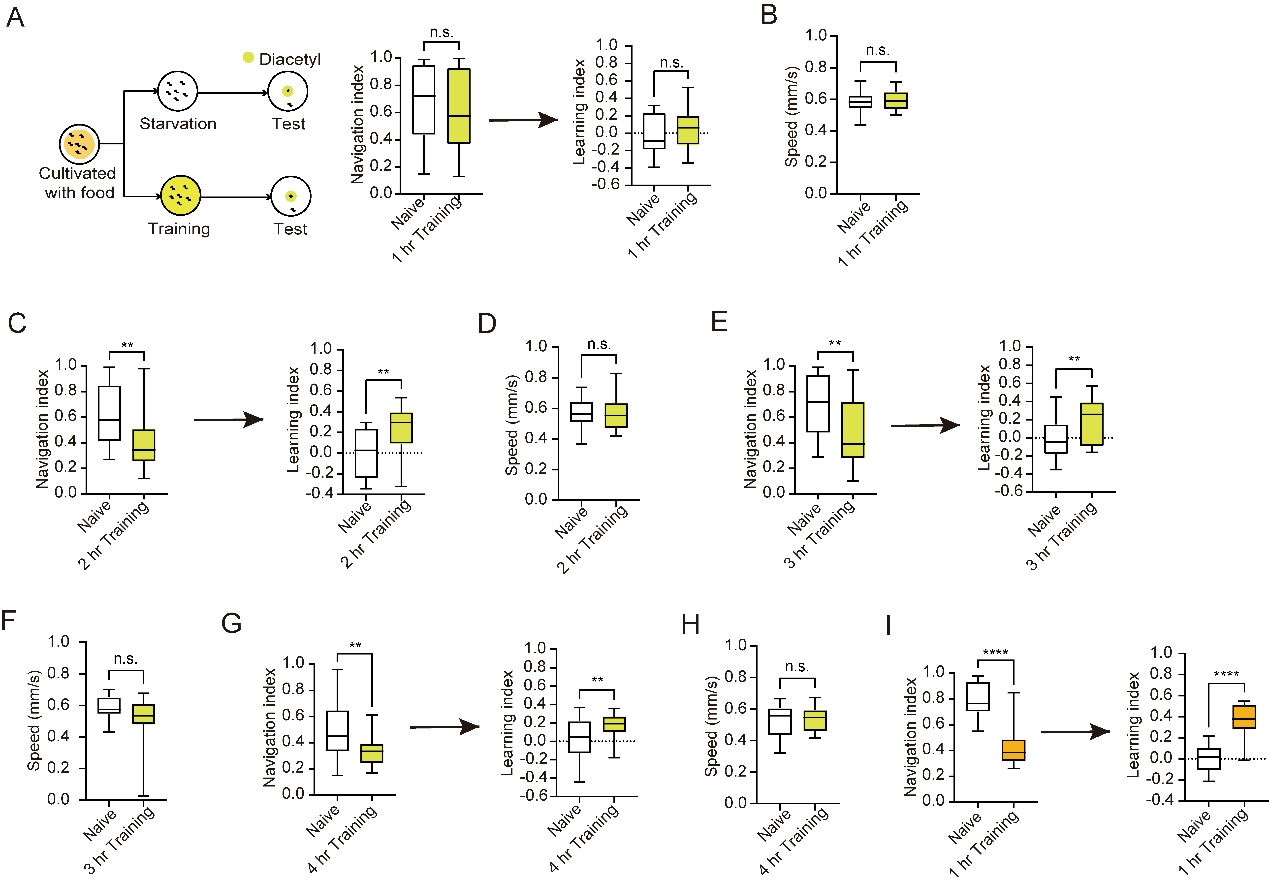


**Supplementary Figure 2. The formation of aversive memory in C. elegans.**

1. Left: Schematic of the aversive olfactory learning paradigm. Middle: Navigation index of naive and 1-hour trained animals with 1:1,0000 diluted diacetyl. Right: Learning index of naive and 1-hour trained animals according to the navigation index (Naive, n = 23; 1hr Training, n = 22). (B) Statistical comparison of speed, between naive (n = 23) and 1-hour trained (n = 22) animals. (C, E, G) Statistical comparison of navigation index and learning index between naive animals and those subjected to 2-hour training (C), 3-hour training (E) and 4-hour training (G) (C: Naive, n = 22; 1hr Training, n = 20; E: n = 23 per group; G: Naive, n = 24; 1hr Training, n = 21). (D, F, H) Statistical comparison of speed between naive and those subjected to 2-hour training (D), 3-hour training (F) and 4-hour training (H) (D: Naive, n = 22; 1hr Training, n = 20; F: n = 23 per group; H: Naive, n = 24; 1hr Training, n = 21). (I) Statistical comparison of navigation index and learning index between naive animals and 1-hour trained animals with 1:1,000 diluted diacetyl (n = 16 per group). Data are presented as boxplots showing the maximum value, 75th percentile, median, 25th percentile, and minimum value. Statistical significance was determined using Student's t-test. **P < 0.01; ****P < 0.0001; n.s., not significant.


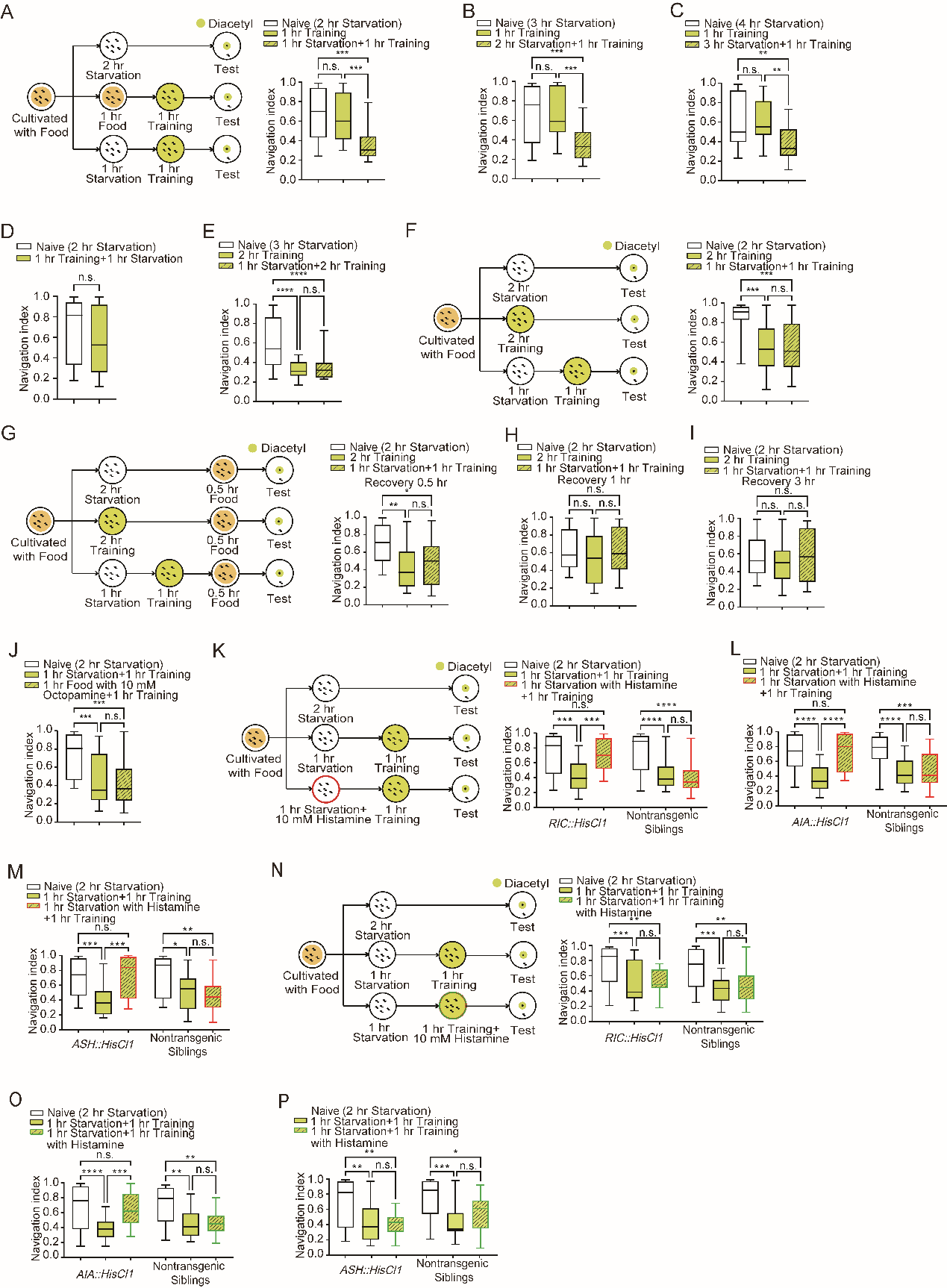


**Supplementary Figure 3. Navigation index during aversive olfactory learning**

1. Schematic representation of the behavioral paradigm for aversive olfactory learning (Left). Statistical comparison of navigation index in naive animals, those immediately after 1-hour training, and those after 1-hour training followed by 1-hour starvation (Right) (n = 22 per group). (B-C) Statistical comparison of navigation index in naive animals, those immediately after 1-hour training, and those after 1-hour training followed by 2-hour (B) (n = 22 per group) and 3-hour starvation (C) (n = 21 per group). (D) Statistical comparison of navigation index between worms subjected to 1-hour starvation followed by 1-hour training and control animals subjected to 2-hour starvation (n = 24 per group). (E) Statistical comparison of navigation index among worms subjected to 2-hour training followed by 1-hour starvation, naive animals, and worms subjected to 2-hour training alone. (F) Schematic representation of the behavioral paradigm and statistical comparison of navigation index among worms subjected to 1-hour training followed by 1-hour starvation (n = 21), naive animals (n = 22), and those subjected to 2-hour training alone (n = 22). (G-I) Schematic representation of the behavioral paradigm for memory retention after 0.5-hour recovery with food. Statistical comparison of navigation index at 0.5 hour (G) (Naive, n = 23; 2hr Training, n = 21; 1hr Starvation + 1hr Training, n = 22), 1 hour (H) (Naive, n = 24; 2hr Training, n = 23; 1hr Starvation + 1hr Training, n = 22), and 3 hours (I) (Naive, n = 25; 2hr Training, n = 24; 1hr Starvation + 1hr Training, n = 24) of recovery following 1-hour training after 1-hour starvation and 2-hour training. (J) Navigation index comparison among worms subjected to 1-hour training followed by 1-hour starvation, naive worms and those subjected to 1-hour training followed by exogenous octopamine treatment with food for 1 hour (n = 24 per group). (K) Schematic representation (Left) and statistical comparison (Right) of navigation index for manipulating the RIC neuron with histamine treatment during the starvation session (n = 23 per group). (L) Statistical comparison of navigation index for manipulating the AIA neuron with histamine treatment during the starvation session (n = 23 per group). (M) Statistical comparison of navigation index for manipulating the ASH neuron with histamine treatment during the starvation session (n = 23 per group). (N) Schematic representation (Left) and statistical comparison (Right) of navigation index for manipulating the RIC neuron with histamine treatment during the training session (n = 22 per group). (O) Statistical comparison of navigation index for manipulating the AIA neuron with histamine treatment during the training session (n = 23 per group). (P) Statistical comparison of navigation index for manipulating the ASH neuron with histamine treatment during the training session (n = 21 per group). Data are presented as boxplots showing the maximum value, 75th percentile, median, 25th percentile, and minimum value. Statistical significance was determined using Student's t-test or two-way ANOVA with Tukey’s post hoc test. *P < 0.05; **P < 0.01; ***P < 0.001; ****P < 0.0001; n.s., not significant.


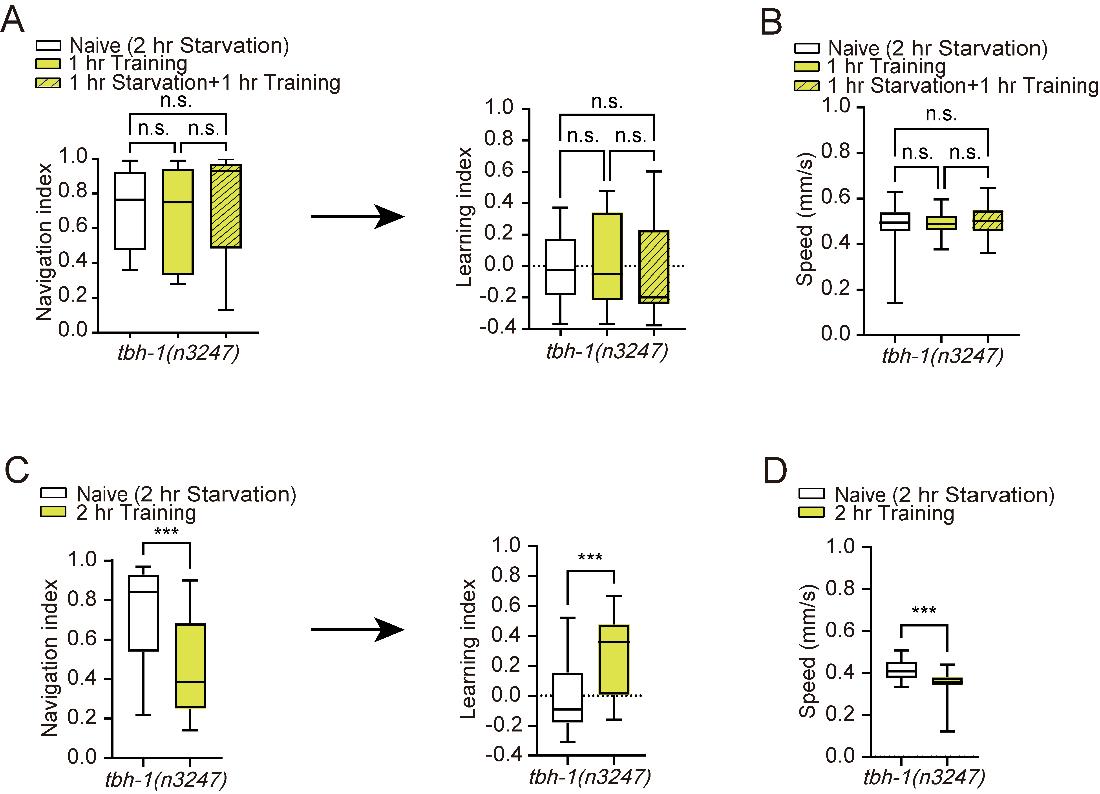


**Supplementary Figure 4. Aversive learning ability in *tbh-1* mutant C. elegans.**

1. Statistical comparison of navigation index and learning index in tbh-1 mutant worms across three conditions: naive animals, animals subjected to 1-hour training only, and animals subjected to 1-hour training followed by 1-hour starvation (n = 24 per group). (B) Statistical comparison of speed in tbh-1 mutant worms across three conditions: naive animals, animals subjected to 1-hour training only, and animals subjected to 1-hour training followed by 1-hour starvation (n = 24 per group). (C) Statistical comparison of navigation index and learning index in tbh-1 mutants between naive and 2-hr aversive training conditions (n = 22 per group). (D) Statistical comparison of peed in tbh-1 mutants between naive and 2-hr aversive training conditions (n = 22 per group). Data are presented as boxplots showing the maximum value, 75th percentile, median, 25th percentile, and minimum value. Statistical significance was determined using Student's t-test or two-way ANOVA with Tukey’s post hoc test. *P < 0.05; ***P < 0.001; ns, not significant.


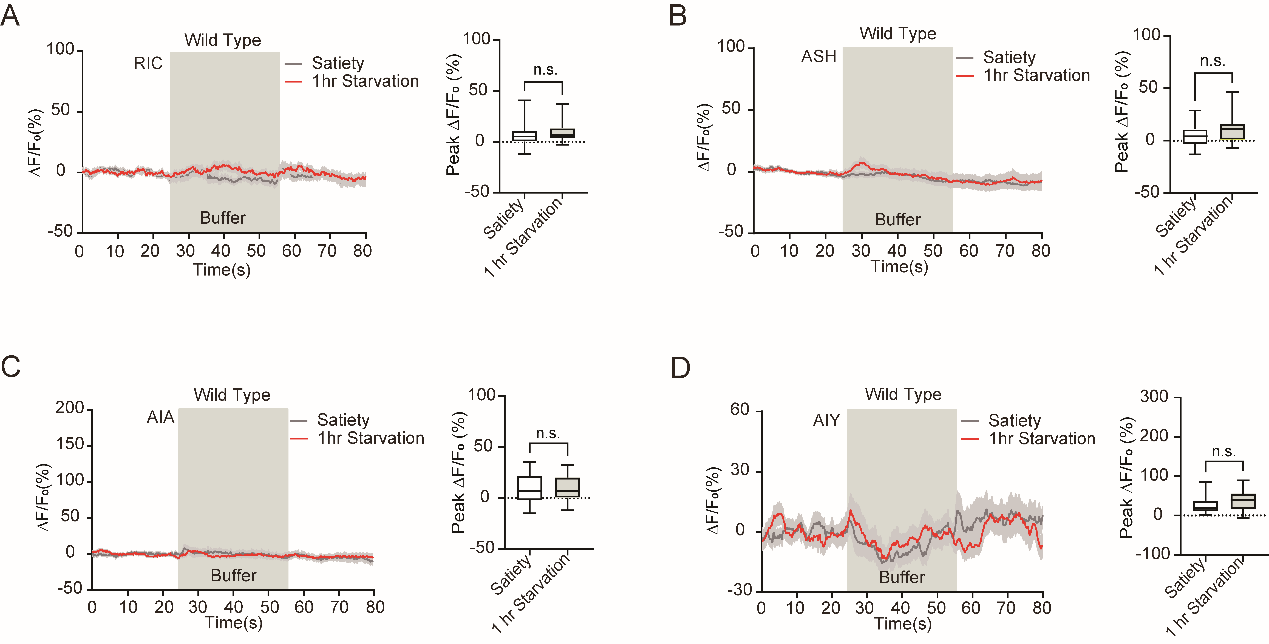


**Supplementary Figure 5. Neuronal responses to shear stress in our microfluidic system**

**(A) RIC neuron does not respond to shear stress in our microfluidic system (n = 14 per group).** (B) **ASH neuron exhibits non-significant response to shear stress in our microfluidic system (**Satiety, n = 12; 1hr Starvation, n = 14**).** (C) **AIA neuron does not respond to shear stress in our microfluidic system (**Satiety, n = 9; 1hr Starvation, n = 10**).** (D) AIY neuron does not response to shear in our microfluidic system **(**Satiety, n = 19; 1hr Starvation, n = 18**)**. Data are presented as boxplots showing the maximum value, 75th percentile, median, 25th percentile, and minimum value. Statistical significance was determined using Student's t-test. n.s., not significant.


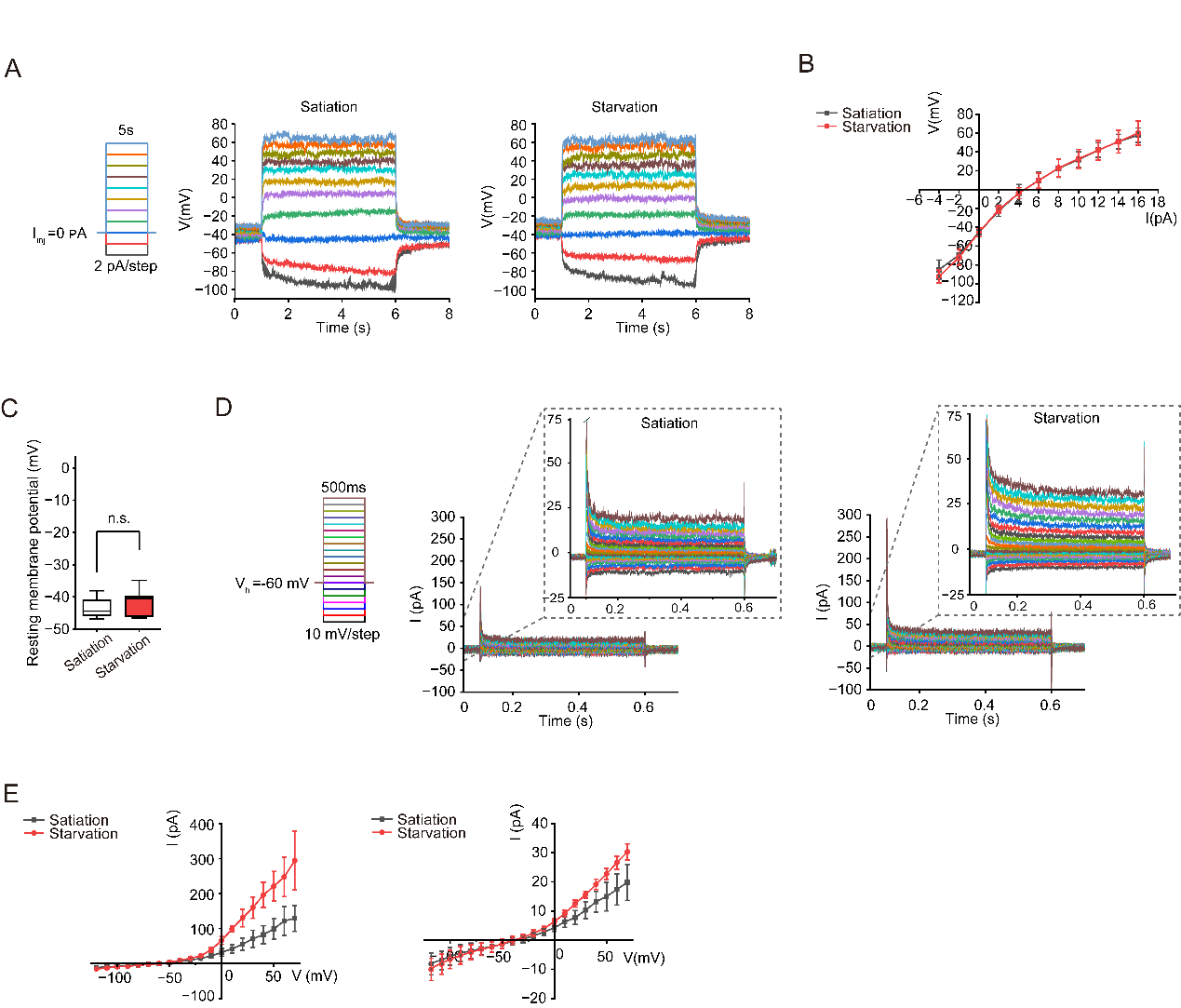


**Supplementary Figure 6. RIC neuron electrophysiological property**

(A) Individual representative membrane potential changes of RIC neurons following different current injection steps in satiated and starved animals. (B) V-I curves under satiation and starvation conditions **(n = 4 per group)**. (C) The resting membrane potential of RIC neurons is similar in satiated **(n = 4)** and starved **(n = 5)** animals. (D) Averaged current changes following different voltage steps under satiation **(n = 6)** and starvation **(n = 5)** conditions. (E) I-V curves of peak current (Left) and I-V curves of steady-state current (Right) under satiation and starvation conditions **(n = 4 per group)**. Data are presented as boxplots showing the maximum value, 75th percentile, median, 25th percentile, and minimum value. Statistical significance was determined using Student's t-test. n.s., not significant.


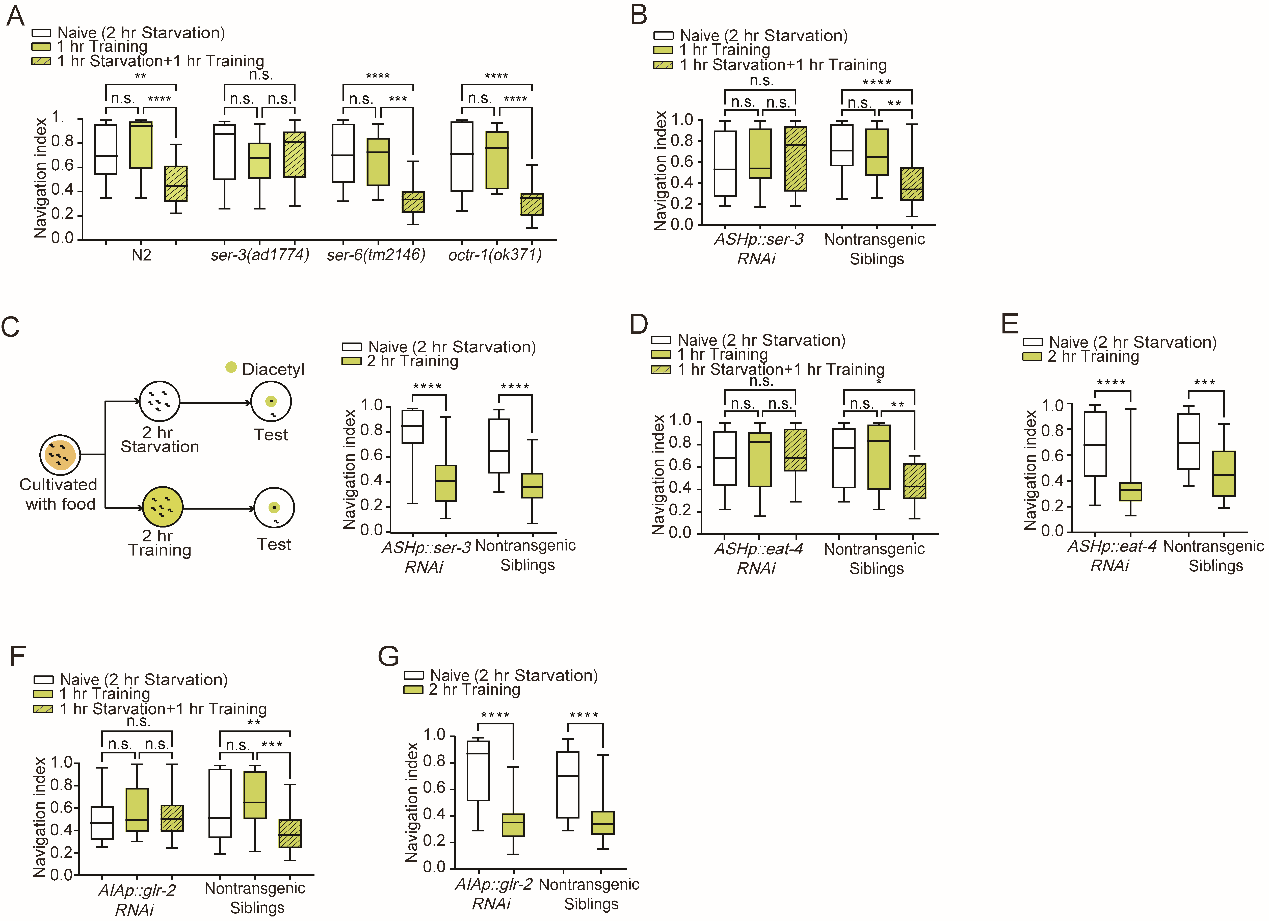


**Supplementary Figure 7. Navigation index during aversive olfactory learning in mutant and transgenic *C. elegans* strains**

1. Statistical comparison of navigation index in octopamine receptor mutants under three conditions: naive, after 1-hour training, and after 1-hour training followed by 1-hour starvation (wild type: n = 20 per group; ser-3: n = 20 per group; ser-6: n = 18 per group, octr-1: Naive, n = 19; 1hr Training, n = 19; 1hr Starvation + 1hr Training, n = 17). (B) Statistical comparison of navigation index in *ASH::ser-3 RNAi* animals and nontransgenic siblings under three conditions: naive, after 1-hour training, and after 1-hour training followed by 1-hour starvation (n = 23 per group). (C) Schematic representation (Left) and statistical comparison (Right) of navigation index for *ASH::ser-3 RNAi* animals under naive and 2 hr training condition (n = 21 per group). (D) Statistical comparison of the navigation index in *ASH::eat-4 RNAi* animals and nontransgenic siblings under three conditions: naive, after 1-hour training, and after 1-hour training followed by 1-hour starvation (*ASHp::eat-4 RNAi*: n = 23 per group; Nontransgenic Siblings: Naive, n = 20; 1hr Training, n = 20; 1hr Starvation + 1hr Training, n = 21). (E) Statistical comparison of navigation index for *ASH::eat-4 RNAi* animals nontransgenic siblings under naive and 2 hr training condition(*ASHp::eat-4 RNAi*: n = 23 per group; Nontransgenic Siblings: n = 22 per group). (F) Statistical comparison of navigation index in *AIA::glr-2 RNAi* animals and nontransgenic siblings under three conditions: naive, after 1-hour training, and after 1-hour training followed by 1-hour starvation (*AIAp::glr-2 RNAi*: Naive, n = 22; 1hr Training, n = 23; 1hr Starvation + 1hr Training, n = 23; Nontransgenic Siblings: n = 23 per group). (G) Statistical comparison of navigation index for *AIA::glr-2 RNAi* animals nontransgenic siblings under naive and 2 hr training condition (n = 23 per group). Data are presented as boxplots showing the maximum value, 75th percentile, median, 25th percentile, and minimum value. Statistical significance was determined using Student's t-test or two-way ANOVA with Tukey’s post hoc test. *P < 0.05; **P < 0.01; ***P < 0.001; ****P < 0.0001; n.s., not significant.


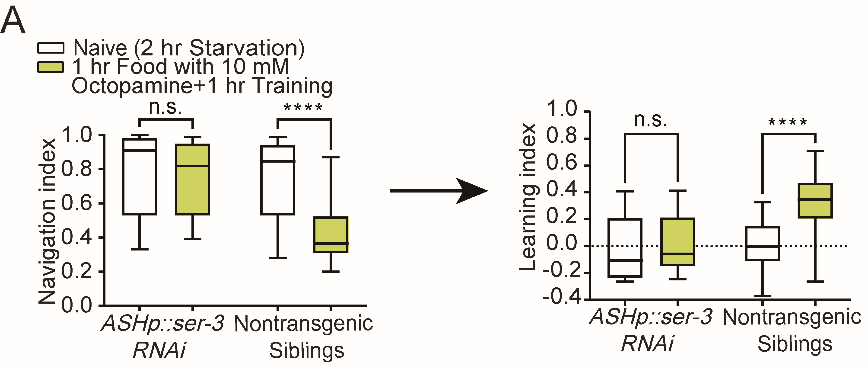


**Supplementary Figure 8. Suppression of *ser-3* in ASH neurons attenuates the octopamine-induced enhancement of aversive learning.**

1. Statistical comparison of the navigation index and learning index in naive *ASH::ser-3 RNAi* animals and those subjected to 1-hour training followed by 1-hour octopamine treatment in the presence of food (*ASHp::ser-3 RNAi*: n = 23 per group; Nontransgenic Siblings: n = 22 per group). Data are presented as boxplots showing the maximum value, 75th percentile, median, 25th percentile, and minimum value. Statistical significance was determined using Student's t-test. ****P < 0.0001; n.s., not significant.


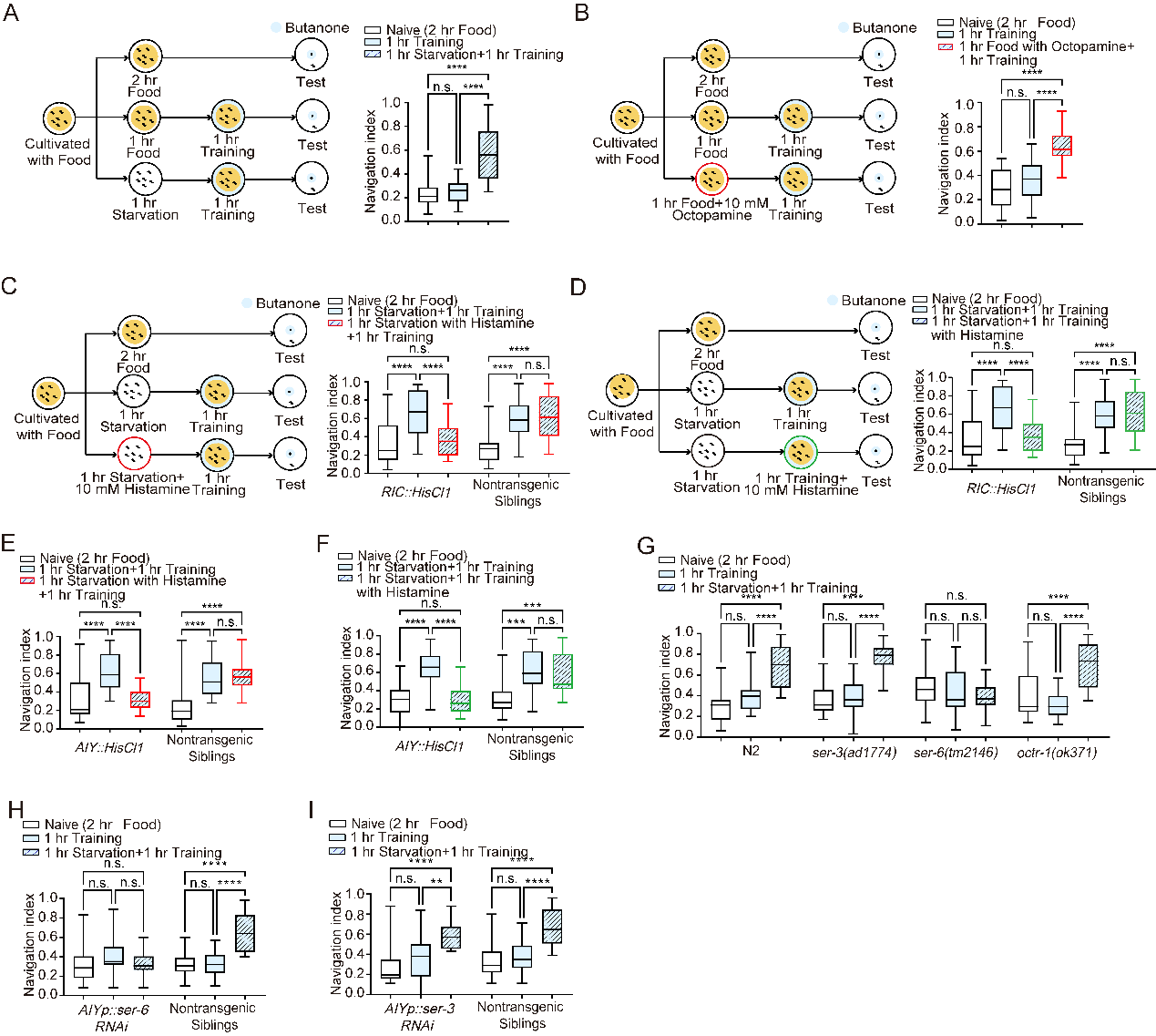


**Supplementary Figure 9. Navigation index during appetitive olfactory learning**

1. Schematic representation of the behavioral paradigm for appetitive olfactory learning (Left). Statistical comparison of navigation index in naive animals (n = 21), those immediately after 1-hour training (n = 19), and those after 1-hour training followed by 1-hour starvation (n = 21) (Right). (B) Statistical comparison for navigation index among worms subjected to 1-hour training followed by 1-hour starvation, naive worms and those subjected to 1-hour training followed by exogenous octopamine treatment with food for 1 hour (n = 20 per group). (C) Schematic representation (Left) and statistical comparison (Right) of navigation index for manipulating the RIC neuron with histamine treatment during the starvation session (n = 21 per group). (D) Schematic representation (Left) and statistical comparison (Right) of navigation index for manipulating the RIC neuron with histamine treatment during the training session (*RICp::HisCl1*: Naive, n = 20; 1hr Starvation+1hr Training, n = 20; 1hr Starvation+1hr Training with Histamine, n = 19; Nontransgenic Siblings: Naive, n = 19; 1hr Starvation+1hr Training, n = 20; 1hr Starvation+1hr Training with Histamine, n = 20). (E) Statistical comparison (Right) of navigation index for manipulating the AIY neuron with histamine treatment during the starvation session (*AIYp::HisCl1*: n = 20 per group; Nontransgenic Siblings: n = 19 per group). (F) Statistical comparison (Right) of navigation index for manipulating the AIY neuron with histamine treatment during the training session (*RICp::HisCl1*: Naive, n = 18; 1hr Starvation+1hr Training, n = 18; 1hr Starvation+1hr Training with Histamine, n = 17; Nontransgenic Siblings: Naive, n = 17; 1hr Starvation+1hr Training, n = 18; 1hr Starvation+1hr Training with Histamine, n = 18). (G) Statistical comparison of navigation index in octopamine receptor mutants under three conditions: naive, after 1-hour training, and after 1-hour training followed by 1-hour starvation (wild type: Naive: n = 21; 1hr Training: n = 20; 1hr Starvation + 1hr Training: n = 21; ser-3: n = 21 per group; ser-6: n = 21 per group, octr-1: n = 20 per group). (H) Statistical comparison of navigation index in *AIY::ser-6 RNAi* animals and nontransgenic siblings under three conditions: naive, after 1-hour training, and after 1-hour training followed by 1-hour starvation (*AIYp::ser-6 RNAi*: Naive, n = 20; 1hr Training, n = 19; 1hr Starvation+1hr Training, n = 20; Nontransgenic Siblings: Naive, n = 18; 1hr Training, n = 21; 1hr Starvation+1hr Training, n = 21). (I) Statistical comparison of navigation index in *AIY::ser-3 RNAi* animals and nontransgenic siblings under three conditions: naive, after 1-hour training, and after 1-hour training followed by 1-hour starvation (*AIYp::ser-3 RNAi*: Naive, n = 18; 1hr Training, n = 17; 1hr Starvation+1hr Training, n = 18; Nontransgenic Siblings: n = 18 per group). Data are presented as boxplots showing the maximum value, 75th percentile, median, 25th percentile, and minimum value. Statistical significance was determined using two-way ANOVA with Tukey’s post hoc test. **P < 0.01; ***P < 0.001; ****P < 0.0001; n.s., not significant.


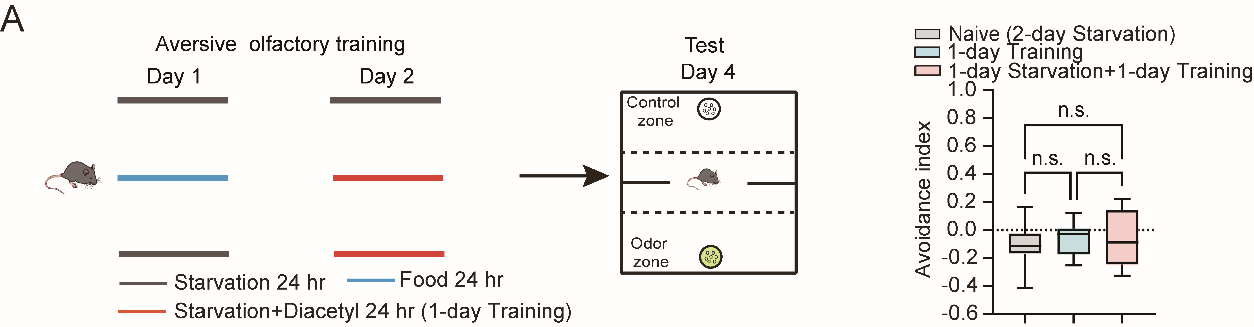


**Figure 10. Norepinephrine signaling regulates hunger-facilitated olfactory learning in mice.**

(A) Schematic representation of the aversive olfactory learning paradigm in mice (Left) and statistical comparison of time spent in the empty chamber under naive (n =13), 1-day training (n = 8), and 1-day training followed by 1-day starvation (n = 13) conditions (Right). Data are presented as boxplots showing the maximum value, 75th percentile, median, 25th percentile, and minimum value. Statistical significance was determined using two-way ANOVA with Tukey’s post hoc test. n.s., not significant.


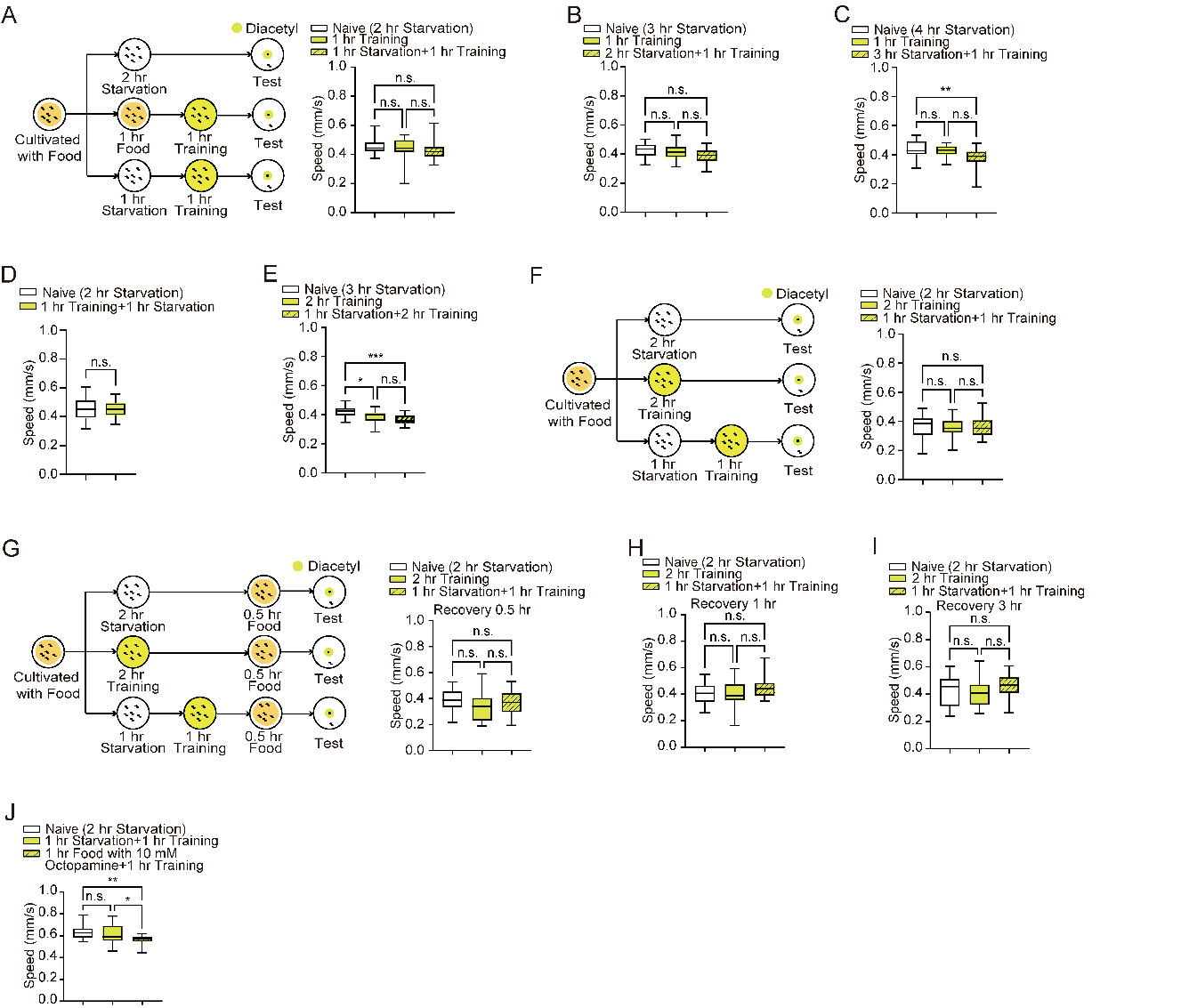


**Supplementary Figure 11. Wild-type worms’ basic locomotive parameters under different aversive training conditions**

1. Schematic representation of the behavioral paradigm for aversive olfactory learning (Left). The speed was assessed in naive animals, those immediately after 1-hour training, and those after 1-hour training followed by 1-hour starvation (n = 22 per group). (B) Statistical comparison of the speed in naive animals, those immediately after 1-hour training, and those after 1-hour training followed by 2-hour starvation (n = 22 per group). (C) Statistical comparison of the speed in naive animals, those immediately after 1-hour training, and those after 1-hour training followed by 3-hour starvation (n = 21 per group). (D) Statistical comparison of the speed in naive animals and those after 1-hour starvation followed by 1-hour training (n = 24 per group). (E) Statistical comparison of the speed in naive animals (n = 22), those immediately after 2-hour training (n = 22), and those after 2-hour training followed by 1-hour starvation (n = 21). (F) Statistical comparison of the speed in naive animals (n = 27), those immediately after 2-hour training (n = 27), and those after 1-hour training followed by 1-hour starvation (n = 25). (G-I) Statistical comparison of the speed, body-bending amplitude and body-bending period at 0.5 hour (G) (Naive, n = 23; 2hr Training, n = 21; 1hr Starvation + 1hr Training, n = 22), 1 hour (H) (Naive, n = 24; 2hr Training, n = 23; 1hr Starvation + 1hr Training, n = 22), and 3 hours (I) of recovery following 1-hour training after 1-hour starvation and 2-hour training (Naive, n = 25; 2hr Training, n = 24; 1hr Starvation + 1hr Training, n = 24). (J) The speed comparison between worms subjected to 1-hour training followed by 1-hour starvation and those subjected to 1-hour training followed by exogenous octopamine treatment with food for 1 hour (n = 24 per group). Data are presented as boxplots showing the maximum value, 75th percentile, median, 25th percentile, and minimum value. Statistical significance was determined using Student's t-test or two-way ANOVA with Tukey’s post hoc test. *P < 0.05; **P < 0.01; ***P < 0.001; n.s., not significant.


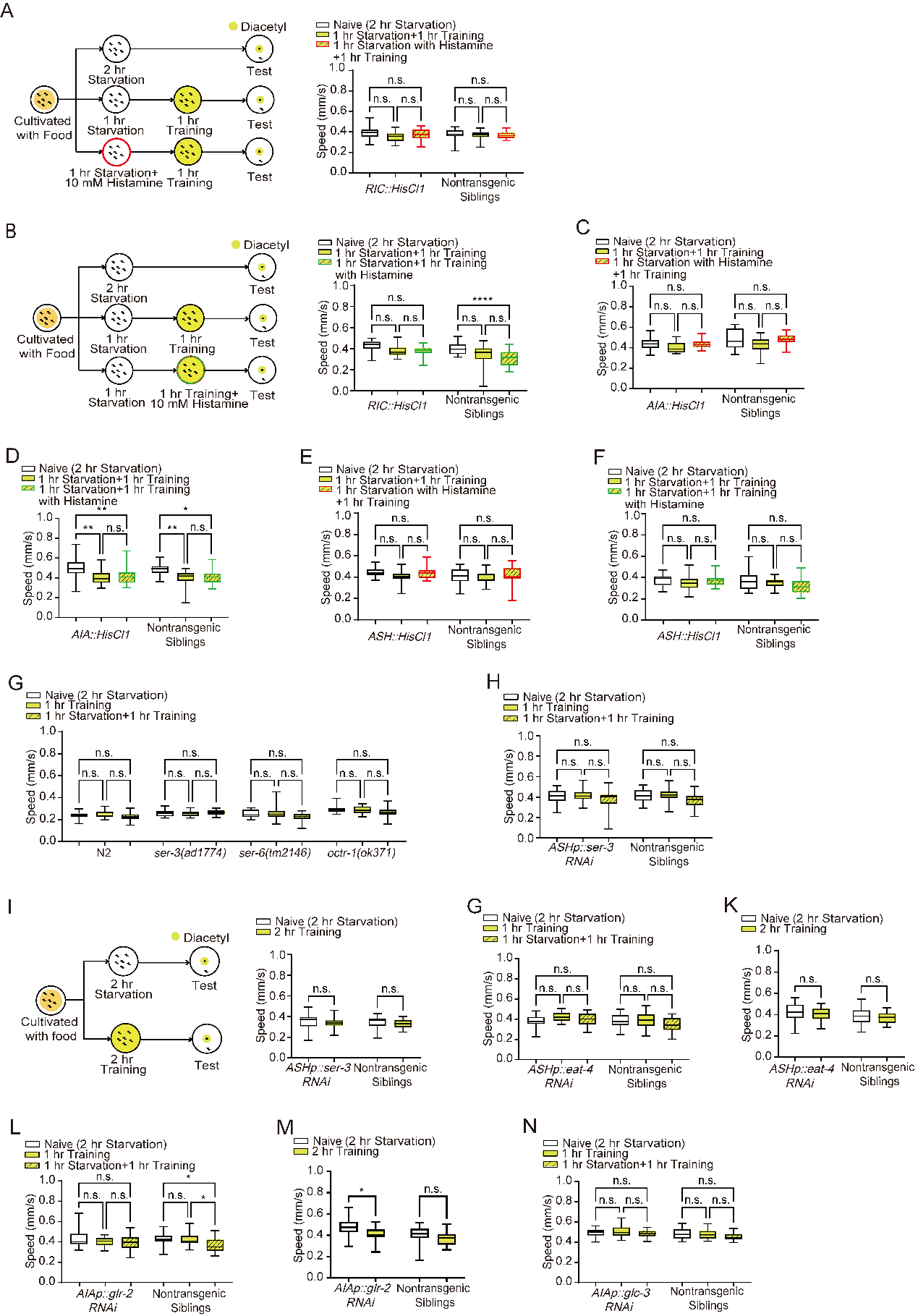


**Supplementary Figure 12. Transgenic, mutant and RNAi strain worms’ basic locomotive speed under different aversive training conditions**

1. Schematic representation (Left) and statistical comparison (Right) of the speed for manipulating the RIC neuron with histamine treatment during the starvation session (n = 23 per group). (B) Schematic representation (Left) and statistical comparison (Right) of the speed for manipulating the RIC neuron with histamine treatment during the training session (n = 22 per group). (C) Statistical comparison of the speed for manipulating the AIA neuron with histamine treatment during the starvation session (n = 23 per group). (D) Statistical comparison of the speed for manipulating the AIA neuron with histamine treatment during the training session (n = 23 per group). (E) Statistical comparison of the speed for manipulating the ASH neuron with histamine treatment during the starvation session (n = 23 per group). (F) Statistical comparison of the speed for manipulating the ASH neuron with histamine treatment during the training session (n = 21 per group). (G) Statistical comparison of the speed in octopamine receptor mutants under three conditions: naive, after 1-hour training, and after 1-hour training followed by 1-hour starvation(wild type: n = 20 per group; ser-3: n = 20 per group; ser-6: n = 18 per group, octr-1: Naive, n = 19; 1hr Training, n = 19; 1hr Starvation + 1hr Training, n = 17). (H) Statistical comparison of the speed in *ASH::ser-3 RNAi* animals and nontransgenic siblings under three conditions: naive, after 1-hour training, and after 1-hour training followed by 1-hour starvation (n = 23 per group). (I) Statistical comparison of the speed for *ASH::ser-3 RNAi* animals under naive and 2 hr training condition (n = 21 per group). (G) Statistical comparison of the speed for *ASH::eat-4 RNAi* animals under three conditions: naive, after 1-hour training, and after 1-hour training followed by 1-hour starvation (*ASHp::eat-4 RNAi*: n = 23 per group; Nontransgenic Siblings: Naive, n = 20; 1hr Training, n = 20; 1hr Starvation + 1hr Training, n = 21). (K) Statistical comparison of the speed, body-bending amplitude and body-bending period for *ASH::eat-4 RNAi* animals under naive and 2 hr training condition (*ASHp::eat-4 RNAi*: n = 23 per group; Nontransgenic Siblings: Naive, n = 20; 1hr Training, n = 20; 1hr Starvation + 1hr Training, n = 21). (L) Statistical comparison of the speed for *AIA::glr-2 RNAi* animals under three conditions: naive, after 1-hour training, and after 1-hour training followed by 1-hour starvation (*AIAp::glr-2 RNAi*: Naive, n = 22; 1hr Training, n = 23; 1hr Starvation + 1hr Training, n = 23; Nontransgenic Siblings: n = 23 per group). (M) Statistical comparison of the speed for *AIA::glr-2 RNAi* animals under naive and 2 hr training condition (n = 23 per group). (N) Statistical comparison of the speed for *AIA::glc-3 RNAi* animals under three conditions: naive, after 1-hour training, and after 1-hour training followed by 1-hour starvation (*AIAp::glc-3 RNAi*: n = 23 per group; Nontransgenic Siblings: n = 22 per group). Data are presented as boxplots showing the maximum value, 75th percentile, median, 25th percentile, and minimum value. Statistical significance was determined using two-way ANOVA with Tukey’s post hoc test. *P < 0.05; **P < 0.01; n.s., not significant.


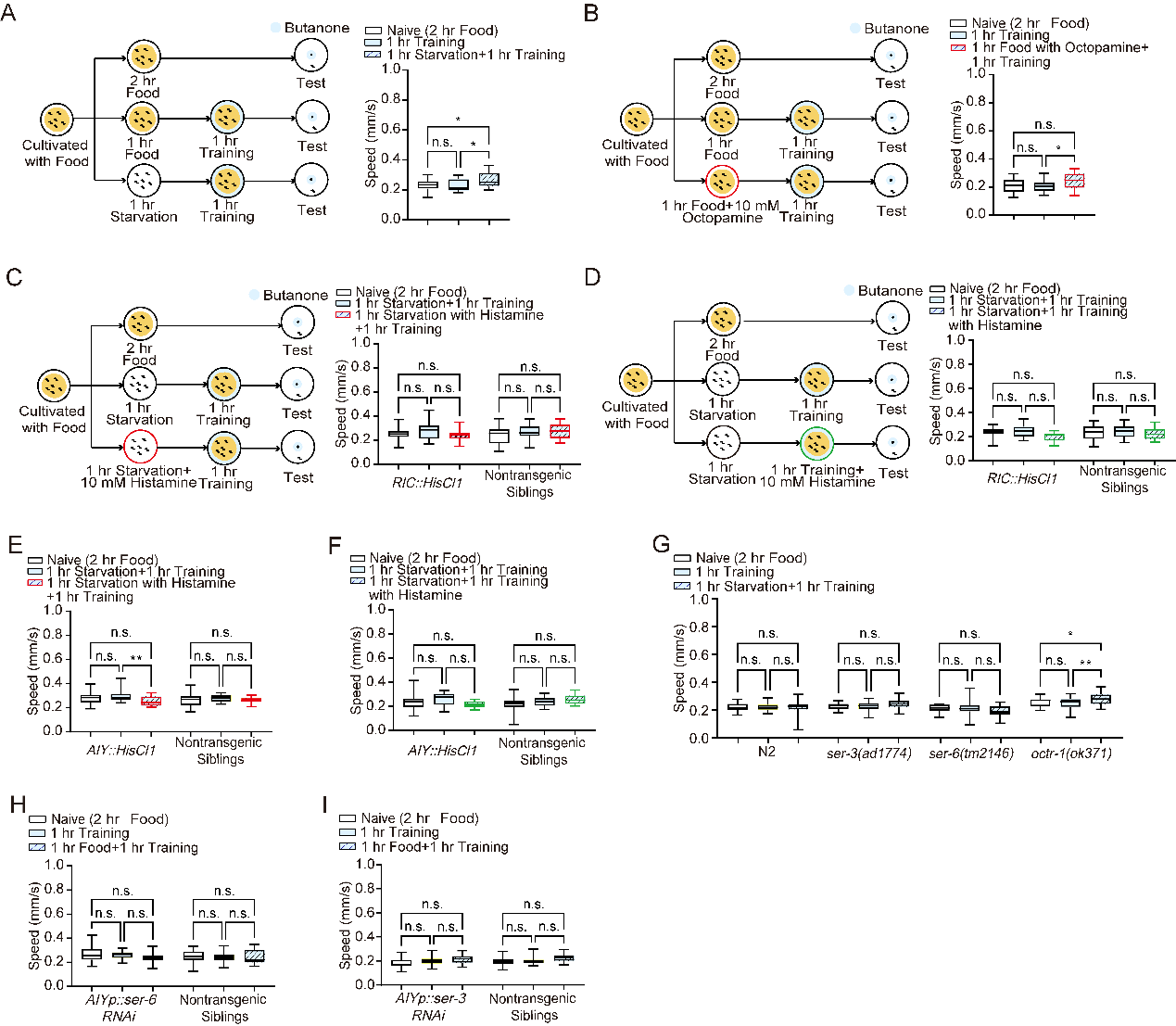


**Supplementary Figure 13. Wild-type and transgenic, mutant and RNAi strain worms’ basic locomotive speed under different appetitive training conditions**

1. The speed was assessed in naive animals (n = 21), those immediately after 1-hour training (n = 21), and those after 1-hour training followed by 1-hour starvation (n = 19). **(B)** The speed comparison between worms subjected to 1-hour training followed by 1-hour starvation and those subjected to 1-hour training followed by exogenous octopamine treatment with food for 1 hour (n = 20 per group). **(C)** Statistical comparison of the speed for manipulating the RIC neuron with histamine treatment during the starvation session (n = 21 per group). **(D)** Statistical comparison of the speed for manipulating the RIC neuron with histamine treatment during the training session (*RICp::HisCl1*: Naive, n = 20; 1hr Starvation+1hr Training, n = 20; 1hr Starvation+1hr Training with Histamine, n = 19; Nontransgenic Siblings: Naive, n = 19; 1hr Starvation+1hr Training, n = 20; 1hr Starvation+1hr Training with Histamine, n = 20). **(E)** Statistical comparison of the speed for manipulating the AIY neuron with histamine treatment during the starvation session (*AIYp::HisCl1*: n = 20 per group; Nontransgenic Siblings: n = 19 per group). **(F)** Statistical comparison of the speed for manipulating the AIY neuron with histamine treatment during the training session (*AICY::HisCl1*: Naive, n = 18; 1hr Starvation+1hr Training, n = 18; 1hr Starvation+1hr Training with Histamine, n = 17; Nontransgenic Siblings: Naive, n = 17; 1hr Starvation+1hr Training, n = 18; 1hr Starvation+1hr Training with Histamine, n = 18). (G) Statistical comparison of the speed in octopamine receptor mutants under three conditions: naive, after 1-hour training, and after 1-hour training followed by 1-hour starvation (wild type: n = 20 per group; ser-3: n = 20 per group; ser-6: n = 18 per group, octr-1: Naive, n = 19; 1hr Training, n = 19; 1hr Starvation + 1hr Training, n = 17). (H) Statistical comparison of the speed in *AIY::ser-6 RNAi* animals and nontransgenic siblings under three conditions: naive, after 1-hour training, and after 1-hour training followed by 1-hour starvation (*AIYp::ser-6 RNAi*: Naive, n = 20; 1hr Training, n = 19; 1hr Starvation+1hr Training, n = 20; Nontransgenic Siblings: Naive, n = 18; 1hr Training, n = 21; 1hr Starvation+1hr Training, n = 21). (I) Statistical comparison of the speed in *AIY::ser-3 RNAi* animals and nontransgenic siblings under three conditions: naive, after 1-hour training, and after 1-hour training followed by 1-hour starvation (*AIYp::ser-3 RNAi*: Naive, n = 18; 1hr Training, n = 17; 1hr Starvation+1hr Training, n = 18; Nontransgenic Siblings: n = 18 per group). Data are presented as boxplots showing the maximum value, 75th percentile, median, 25th percentile, and minimum value. Statistical significance was determined using two-way ANOVA with Tukey’s post hoc test. *P < 0.05; **P < 0.01; n.s., not significant.
